# IL-22 and IL-23 regulate the anticryptococcal response during *Cryptococcus deuterogattii* infection

**DOI:** 10.1016/j.isci.2024.111054

**Published:** 2024-09-27

**Authors:** Israel Diniz-Lima, Ariel Gomes, Mayck Medeiros, Joyce Cristina Guimarães-de-Oliveira, Idália Maria Ferreira-dos-Santos, Elias Barbosa da Silva-Junior, Alexandre Morrot, Danielle Oliveira Nascimento, Leonardo Freire-de-Lima, Lycia de Brito-Gitirana, Fernanda Ferreira Cruz, Debora Decote-Ricardo, Herbert Leonel de Matos Guedes, Celio Geraldo Freire-de-Lima

**Affiliations:** 1 Instituto de Biofísica Carlos Chagas Filho, Universidade Federal do Rio de Janeiro, Rio de Janeiro 21941-900, Brazil; 2 Instituto Oswaldo Cruz, FIOCRUZ, Rio de Janeiro 21045-900, Brazil; 3 School of Medicine, Tuberculosis Research Center, Federal University of Rio de Janeiro, Rio de Janeiro 21941-909, Brazil; 4 Instituto de Veterinária, Universidade Federal Rural do Rio de Janeiro, Seropédica 23890-000, Brazil; 5 Instituto de Ciências Biomédicas, Universidade Federal do Rio de Janeiro, Rio de Janeiro 21941-900, Brazil; 6 Instituto de Microbiologia Paulo de Góes, Universidade Federal do Rio de Janeiro, Rio de Janeiro 21941-900, Brazil

**Keywords:** Pathophysiology, Disease, Immunology

## Abstract

Cryptococcosis is a neglected fungal disease that causes many deaths annually, is primarily caused by *Cryptococcus neoformans* and *Cryptococcus gattii* species. They are environmental fungus that engages lung pneumonia and a severe systemic infection. The rising incidence of affected immunocompetent hosts, particularly by the aggressive *Cryptococcus deuterogattii* (R265), underscores the urgency to understand factors influencing its dissemination. The immunopathogenesis of R265 infection is incompletely understood. Therefore, we investigate the role of IL-22 and IL-23 cytokines during R265 cryptocococcosis. Our findings highlight the crucial role of IL-22 and IL-23 cytokines in lung barrier homeostasis, preventing excessive lung damage. IL-22 not only prevents neutrophil infiltration and IL-17A production but also facilitates eosinophil lung infiltration. Ultimately, this study contributes vital insights into the selective role of IL-22 and IL-23 cytokines in immune activation and tissue regulation during the aggressive R265 lung and systemic infection.

## Highlights

• *Cryptococcus deuterogattii* increases IL-22 in the lung and brain of infected mice
• IL-22 absence during infection leads to less yeast titanization in the lungs
• IL-22 deficiency enhances lung inflammation and eosinopenia during lung infection
• IL-22 deficiency speeds early lung function loss during *C. deuterogattii* infection

## Graphical abstract

**Figure:**
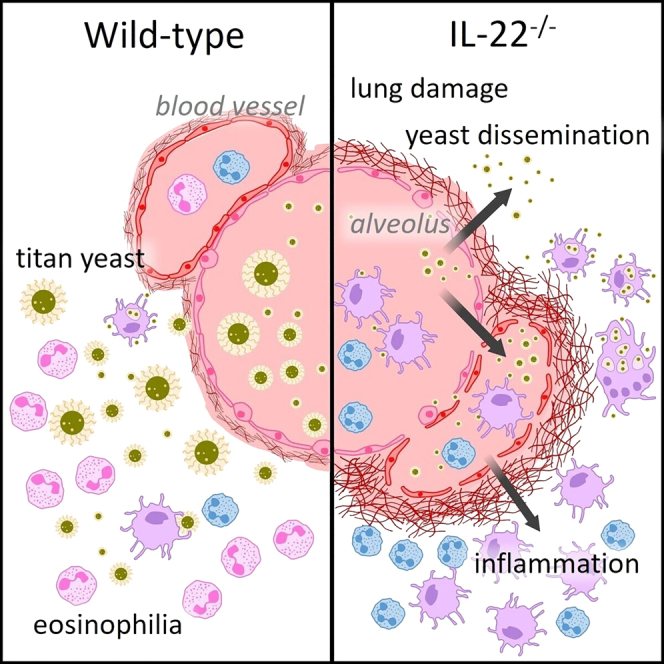

## Introduction

Cryptococcosis is a concerning fungal disease that causes more than 200 thousand deaths annually.^1^ The main culprits behind this disease are the major pathogens known as *Cryptococcus neoformans* and *Cryptococcus gattii* species, both environmental fungi worldwide distributed and restricted to environmental to host dispersion.^2^ The infection starts when environmental spores are in contact with the airway system in mammal hosts.^3^ A variety of risk factors could lead to susceptibility of cryptococcosis, such as diabetes,^4^ prolonged cancer treatment,^5^ chronical use of corticosteroids,^6^ pulmonary chronic illness,^7^ and smoking habits,^8^ but the main risk factor is conditioned by a low immune competence.^9^ Although *C. neoformans* is an opportunistic pathogen that infects mainly immunocompromised hosts, *C. gattii* species causes a more aggressive form of cryptococcosis that origins from a primary infection in immunocompetent individuals.^10^ Nevertheless, an increasing number of *C. gattii* infections over the years has reaching concerning importance, as seen by newly reported cases of these species causing infection in areas previously not associated with its incidence.^11^ Furthermore, immunocompetent hosts are increasingly being afflicted by cryptococcosis, in some cases, around 60% of the infected hosts are immunocompetent and 67% associated with cryptococcal meningoencephalitis.^12^^,^^13^ This scenario in worsened by the fact that more aggressive species of *C. gattii* complex has taken a broad distribution worldwide, such is the case for *Cryptococcus deuterogattii*, correlated with the VGII genotype known as R265.^14^ This species is becoming predominantly between other *C. gattii* species around the globe.^15^^,^^16^^,^^17^ Also, there is limited knowledge regarding the factors favoring R265 dissemination and its impact on immunocompetent hosts. Furthermore, the specific immune system differences that should be conditioning a more aggressive form of cryptococcosis remains unclear.

R265 takes place as a primary infection in the host lungs.^18^ In humans the infection takes over many months to lead into clinical symptoms,^19^ thus turning challenging to diagnosing the disease and restricting the time of treatment.^20^ Therefore, the pulmonary barrier and its microenvironment seems to be decisive to limit fungal dissemination before reaching cryptococcal lung immune evasion.^21^ In this sense, the lung epithelium produces *β*-defensins by IL-22 stimulus that enhances cryptococcal containment in the alveoli space, preserving the epithelium integrity and tissue healing repair.^22^ The role of IL-22 production, a cytokine of the IL-10 family,^23^ is also tied to the immune activation in response to cryptococcal control.^24^ Additionally, it is known for other cryptococcal species that IL-22 production and, at an earlier stage in immune cell activation, the generation of IL-23, are responsible for a better outcome during the disease manifestation.^25^^,^^26^ Both cytokines play important roles during the antifungal immunity, mainly related with Th1 and Th17 antifungal responses and epithelial integrity.^27^

Previous studies have noted a decreased survival rate among IL-23 deficient mice during *C. neoformans* infection.^28^ However, in contrast to *C. neoformans*, R265 elicits a more severe infection, particularly in C57BL/6 mice,^29^ yet the impacts of the IL-23 axis during this infection remain unexplored. The aggressive nature of R265 also diverges from *C. neoformans* in terms of immunopathogenesis. For example, only R265 has been shown to induce dendritic cell phagolysosome paralysis, evading fungal elimination during the anticryptococcal response.^30^ R265, unlike *C. neoformans*, also restricts macrophage activation during infection, resulting in decreased IL-12 release and IFN-γ response.^31^ However, since macrophage activation is pivotal for cryptococcal control and R265 hampers phagocyte activation, the reduced dissemination to the brain compared to *C. neoformans* is associated with less pronounced IFN-γ responses.^32^ Consequently, this affects the progression of systemic infection, as R265 is more confined to the lungs, exhibiting lower fungemia compared to *C. neoformans* infection in C65BL/6 mice.^33^ Additionally, R265 infection can attenuate Th1 and Th17 responses, limiting phagocyte activation and neutrophil recruitment during lung infection.^34^ Notably, unlike *C. neoformans*, R265 also induces a more pro-inflammatory cytokine profile and IL-22 production,^35^ thereby modulating the lung microenvironment differently than other cryptococcal infections. Based on these observations, we hypothesize that R265 infection disrupts lung anticryptococcal immunity by modulating lung cell infiltration and airway-blood barrier homeostasis through IL-23 and IL-22 dependent mechanisms. Therefore, here we describe important implications concerning the roles of IL-22 cytokine during R265 experimental infection.

Employing a mouse infection model, we assess the survival of animals using gene knockout mice for the IL-23 and IL-22 cytokines. Additionally, we also examine other immunological and histopathological features. Our data show that IL-23 and IL-22 are decisive for lung barrier homeostasis, limiting fungal systemic dissemination, and preventing excessive lung damage. Moreover, IL-22 also limits neutrophilic infiltration and excessive Th17 responses. Furthermore, IL-22 is correlated with the R265-induced eosinophilia and a prompter Th2 profile activation. In summary, we describe a selective role for IL-22 immune activation and tissue regulation properties during the aggressive R265 lung and systemic infection.

## Results

### IL-22 deficiency heightens susceptibility to *Cryptococcus deuterogattii* infection

**Figure 1:**
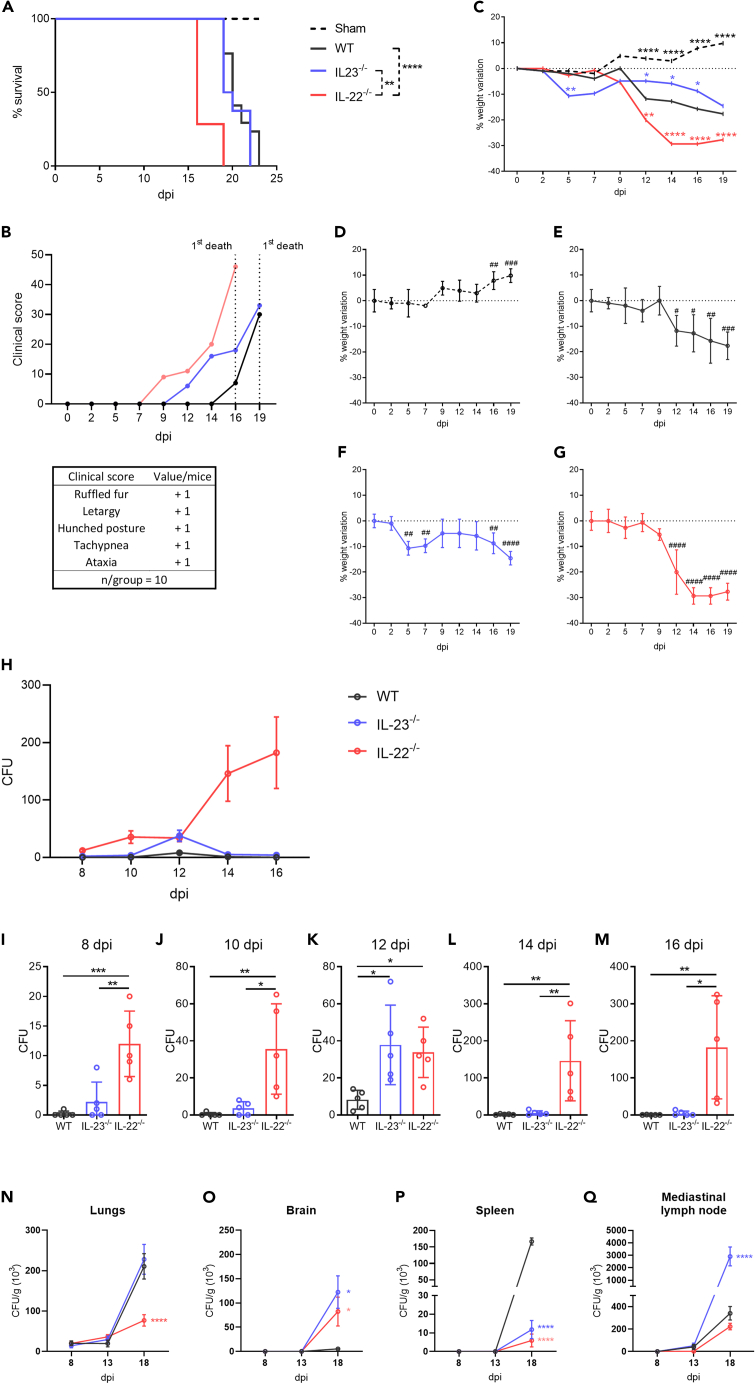
Clinical and pathological analysis of R265 infection Wild type (WT) (*n* = 10), IL-23^−/−^ (*n* = 10), and IL-22^−/−^ (*n* = 10) C57BL/6 mice were intratracheally (i.t.) infected with 10^4^ R265 yeast and observed over time. (A) Survival was monitored for 25 days, and data were presented as percentages. (B) The clinical features were assessed through a sum of scores for each animal disposed on the square. Analysis was stopped when the first animal from each group died. (C–G) Animals were also weighed weekly, two to three times for 19 days, and data are presented as percentages. (C) All groups together. (D) Sham group curve, (E) WT group curve, (F) IL-23^−/−^ group curve, and (G) IL-22^−/−^ group curve. (H–Q) Fungemia in mice was assessed in blood samples collected from the tail vein and directly plated onto Sabouraud-agar culture plates to count colony forming units (CFU) 4 days later. Analysis was performed every two days, starting from 8 dpi to 16 dpi. (H) Fungemia kinetics during the infection. (I, J, K, L, and M) fungemia on days 8, 10, 12, 14, and 16, respectively. (N–Q) Animals had their organs surgically collected after euthanasia at 8 dpi, 13 dpi, and 18 dpi and were macerated at different dilutions. Lung samples were diluted at 1:10000, while other organs were diluted at 1:100. Diluted samples were plated onto Sabouraud-agar culture plates for colony-forming units (CFU) to be counted 4 days later. Fungal burden from (N) lung, (O) brain, (P) spleen and (Q) mediastinal lymph node. ∗∗∗∗*p* < 0.0001, ∗∗∗*p* < 0.001, ∗∗*p* < 0.01, ∗*p* < 0.05 for comparison with the WT group. ^# # # #^*p* < 0.0001, ^# # #^*p* < 0.001, ^# #^*p* < 0.01, ^#^*p* < 0.05 for comparison within each group relative to day 0. (C, N–Q) (blue , , ) IL-23^−/−^ and (red , , ) IL-22^−/−^ indicate comparison with WT. Representative data of 3 independent experiments. Data are represented as mean ± SD. (See also Figure S1).

To investigate the impact of IL-22 and IL-23, we evaluated the knockout mice susceptibility to infection. Despite the wild type (WT) and the IL-23^−/−^ mice presented the same survival rate, with the animals dying around 19 days until 23 days post infection (dpi), the IL-22^−/−^ mice presented lower survival with all animals dead before 19 dpi (Figure 1A). The decreased survival observed in IL-22^−/−^ mice was accompanied by an elevated clinical score early in the infection, surpassing even that of the IL-23^−/−^ mice. In contrast, WT mice only exhibited an increase in their clinical score during the second week of infection (Figure 1B). Non-infected animals increased their weight slightly over time, instead of the infected groups that dropped weight mainly in the late days of infection (Figures 1C, 1D, and 1E). The IL-22^−/−^ mice displayed a prominent weight loss in the late days of infection when compared with the WT infected group (Figures 1C and 1G). Interestingly, despite IL-23^−/−^ mice presented similar survival rate to the WT group, they still showcased a less pronounced weight loss along with a higher clinical score (Figures 1B, 1C, and 1F). Nevertheless, this indicates a heightened susceptibility to R265, particularly when IL-22 cytokine is absent.

Additionally, we explored whether the increased susceptibility to R265 in the absence of IL-23 and IL-22 cytokines resulted from an enhanced systemic dissemination of the fungus. It is known that the timing of *Cryptococcus* spp. dissemination from the initial site to establishing a systemic infection is crucial for the disease outcome and is also associated with the severity of pathogenesis in the host.^36^ Therefore, we accessed the mice fungemia investigating each four days the fungal burden of the blood by counting colony forming units (CFU). Interestingly, we observed none or little CFU on the WT fungemia analysis, but only in the 12 dpi the R265 colonies showed a discreet increase in number (Figure 1H). Both IL-22^−/−^ and IL-23^−/−^ groups presented a significant higher fungemia at this time point (12 dpi, Figure 1K). Surprisingly, only the IL-22^−/−^ group exhibited a consistent and increasing level of fungemia throughout the analysis period, starting from the first week of infection (Figures 1I–1M). The fungal load in this group was several times higher than that in the WT and IL-23^−/−^ groups from the second week (Figures 1L and 1M). This suggests that the peak of fungemia, which was possibly linked to fungal barrier evasion in the lungs around 12 dpi, kept uncontrolled throughout the infection in the absence of IL-22.

Because IL-22 cytokine is important for the tissue healing and repair, and in the context of *Cryptococcus* spp. infection is decisive for the airway-blood barrier homeostasis,^25^ our data indicate that possibly those functions are impaired in the IL-22^−/−^ mice during R265 infection. Therefore, we also analyzed not only the fungal burden of the primary site of infection, the lungs, but also in a draining site, the mediastinal lymph nodes (MLN), and more systemically distributed sites such as spleen and the brain. It is evident that when the brain is affected, a prognosis of meningoencephalitis is indicated, and this represents the most unfavorable prognosis for the outcome of the disease.^37^ Because the R265 yeasts were possible evading in considerable quantities through the airway-blood barrier on 12 dpi (Figure 1K). We defined three time points for fungal burden analysis settling the middle time point one day after 12 dpi. Therefore, the 13 dpi was representative for the second week and 5 days before, 8 dpi was for the first week, and 5 days after, 18 dpi for the third and final week. As expected, in the first and the second week after infection there was no significant difference in fungal burden between groups, but on 18 dpi several differences on the fungal burden were observed (Figures 1N–1Q). Unexpectedly, because of the higher fungemia observed on the absence of IL-22 (Figure 1H), the IL-22^−/−^ mice exhibited the lowest lung fungal burden on the third week of infection (Figure 1N), that could be explained by the greater yeast evasion through the airway-blood barrier. Accordingly, this kinetics was followed by a higher brain fungal burden on in IL-22^−/−^ mice (Figure 1O). Different from the IL-22^−/−^ mice, although IL-23^−/−^ mice presented a higher fungal burden in the brain (Figure 1O) the lung fungal burden was also higher (Figure 1N), thus explaining why only the IL-23^−/−^ group presented a higher fungal burden in the lung draining MLN (Figure 1Q). Accordingly, even days earlier on the infection, at 16 dpi, the absence of IL-23 also sustained a higher lung burden (Figure S1). Curiously, the absence alone of both IL-22 and IL-23, resulted in disfavoring a higher fungal burden in the spleen, only observed in the WT group (Figure 1P). The fugal accumulation in the spleen is connected to the yeast cells that have been engulfed by phagocytes within this organ, representing an attempt to control the infection. This process takes place as these cells migrate systematically to lymphoid sites.^38^^,^^39^

### IL-22 and IL-23 deficiency worsen infection-induced lung damage and amplifies cytokine responses in the lungs and brain differently

**Figure 2:**
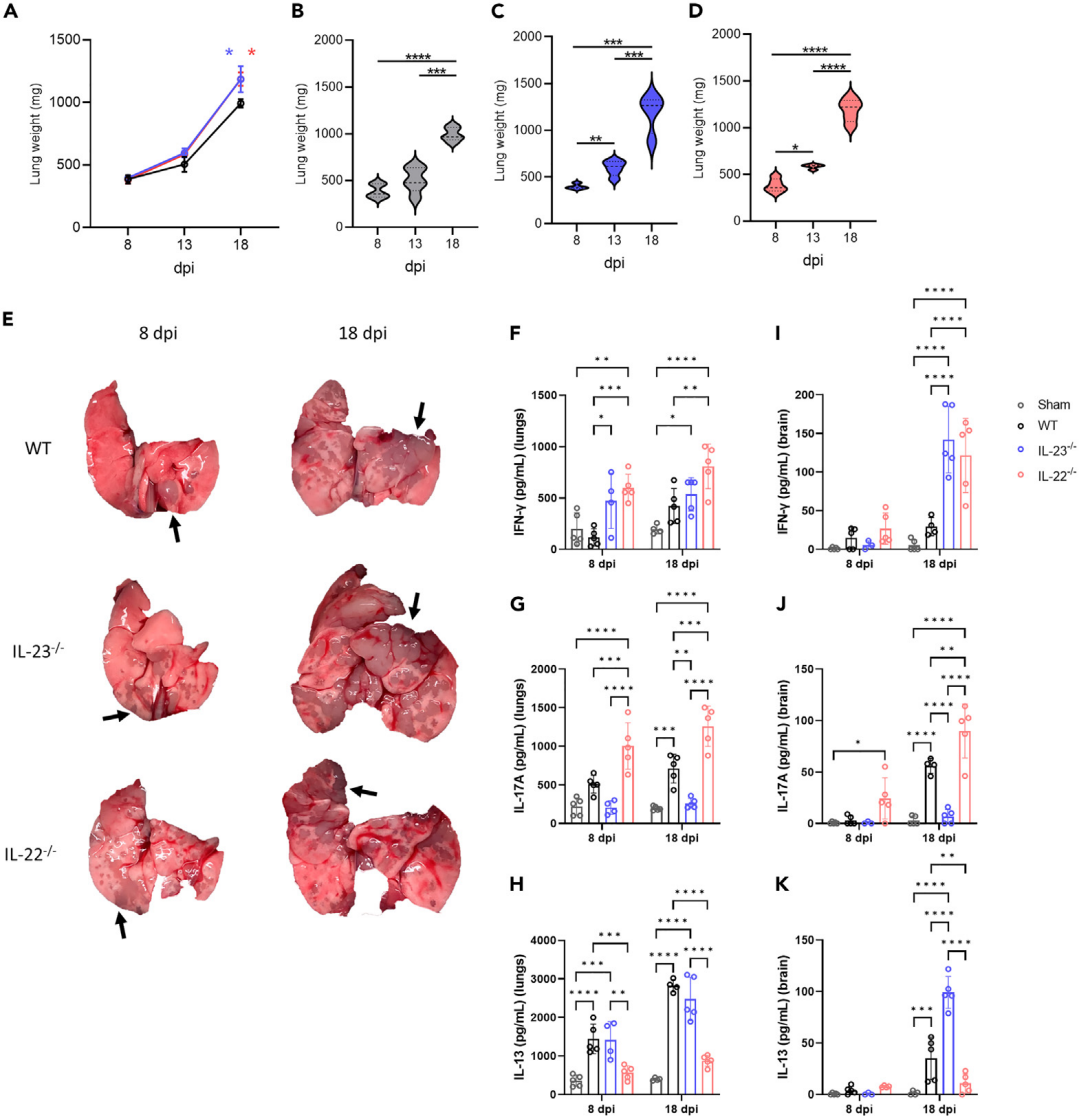
Lung morphology and cytokine analysis of mice experimentally infected with R265 Wild type (WT) (*n* = 5), IL-23^−/−^ (*n* = 5) and IL-22^−/−^ (*n* = 5) C57BL/6 mice were intratracheally (i.t.) infected with 10^4^ R265 yeast and observed over time. Animals had their lungs and brain surgically collected after euthanasia at 8 dpi, 13 dpi, and 18 dpi. (A–D) Lung weighting overtime, (A) for all groups, (B) WT, (C) IL-23^−/−^ and (D) IL-22^−/−^. (E) Representative images of lungs from the 8 and 18 dpi. (↑) Black arrows indicate severely injured lung tissue (grayish areas). Cytokine analysis from (F–H) lungs and (I–K) brain 8 and 18 dpi, (F and I) IFN-γ, (G and J) IL-17A, and (H and K) IL-13 were measured by ELISA. ∗∗∗∗*p* < 0.0001, ∗∗∗*p* < 0.001, ∗∗*p* < 0.01, ∗*p* < 0.05 for comparisons between groups. (A) (blue ) IL-23^−/−^ and (red ) IL-22^−/−^ indicate comparison with WT. Representative data of 2 independent experiments. Data are represented as mean ± SD. (See also Figure S2).

Because of the disparity in fungal burden between the IL-22^−/−^ and IL-23^−/−^, mainly in the primary site, the lungs (Figure 1N), we evaluate the impact of the infection in the lung integrity, and on the cytokine microenvironment of both lungs and brain (Figure 2). The lung weight was increasing overtime for all animals (Figures 2B–2D), but at 18 dpi the absence of IL-22 and IL-23 caused a pronounced increase in weight compared to normal infected animals (Figure 2A). When evaluating the macroscopic morphology of the lungs, is noticeable that at 18 dpi the lungs of all animals were severely injured, but distinctly, at 8 dpi the WT mice exhibited less injured areas when compared to IL-22^−/−^ and IL-23^−/−^ groups (Figure 2E). Hence, it is evident that the inflammatory response in the knockout mice during infection is occurring differently, leading to increased lung damage in the initial stages of the infection. Therefore, we chose to assess the consequences on the cytokine microenvironment in both the lungs and the brain. The Th1 cytokine profile was measured in terms of the IFN-γ production, the Th2 response was evaluated in terms of IL-13 and IL-17A for Th17. Both the lungs and brain had their cytokine microenvironment modulated by the absence of IL-23 and IL-22, mainly in the third week of infection (Figures 2F–2K). The IFN-γ production was several times higher in the knockout groups compared to the WT (Figures 2F and 2I). The lack of IL-23 disfavored the IL-17A production, but the lack of IL-22 accentuated the IL-17A production (Figures 2G and 2J). At 16 dpi during the infection, it became clear that the absence of IL-23 leads to a more Th2 response since it presented the higher levels of IL-4, IL-5, and IL-33 in the lungs, and a marked reduction of the capacity to produce Th17 profile cytokines such as IL-17A and IL-22, and proinflammatory cytokines such as IL-1β and TNF-α (Figure S2). On the contrary, the deficiency of IL-22 led to a marked production of IL-17A, IL-1β, TNF-α, IL-12, and IFN-γ with non-regulatory lung microenvironment with less IL-10 and TGF-β and lower Th2 profile cytokines, such as IL-4, IL-33, and IL-5 (Figure S2). Therefore, a more pronounced Th1/Th17 cytokine axis tends to be favored in the absence of the IL-22 cytokine, and this is reinforced by the fact that the IL-13 production was even lower in those mice, with the opposite happening in the lack of IL-23 (Figures 2H and 2K). Therefore, the Th2 cytokine axis is more prompt to be favored in the lack of IL-23 than IL-22. Furthermore, a more inflammatory lung microenvironment is induced during the infection in the absence of IL-22 cytokine.

### IL-22 deficiency alters lung cell composition, favoring macrophages and neutrophils recruitment while reducing lymphocytes and eosinophils during infection

**Figure 3:**
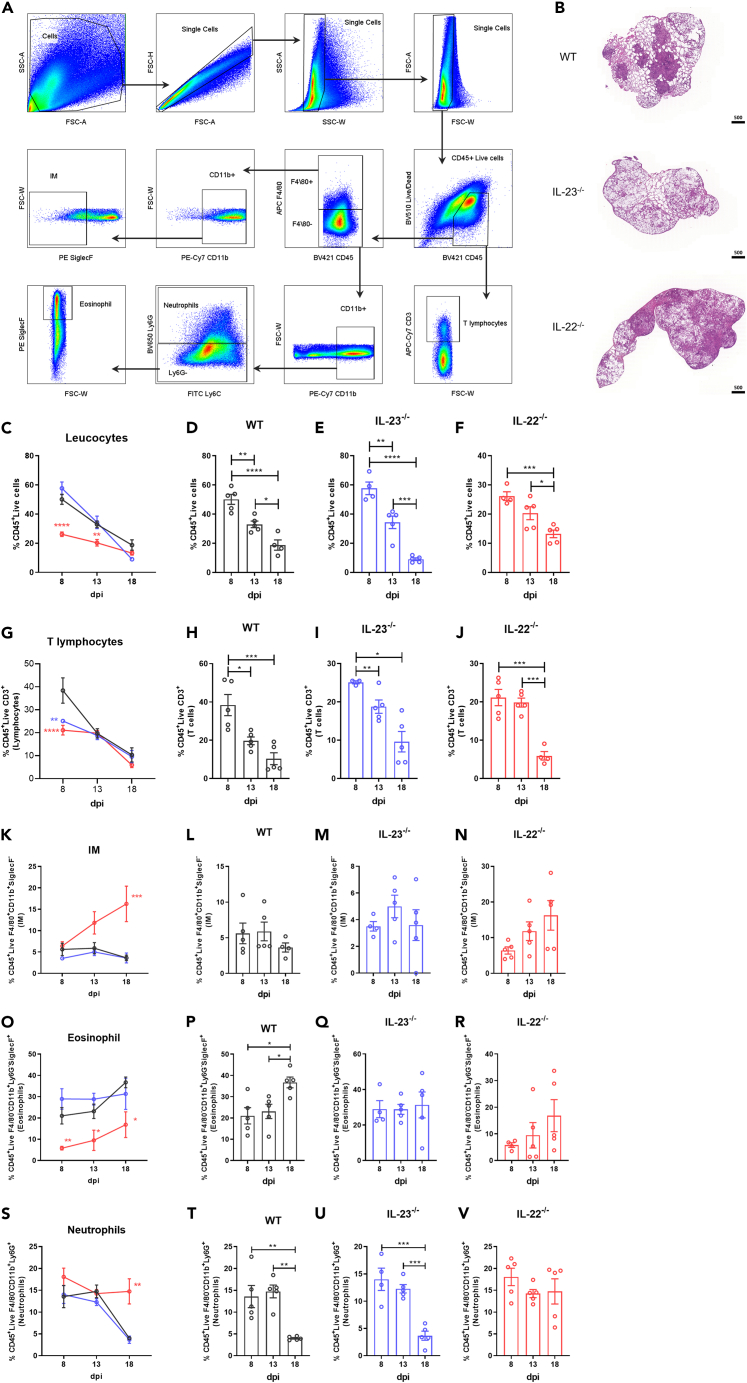
Lung cell population analysis of mice experimentally infected with R265 Wild type (WT) (*n* = 5), IL-23^−/−^ (*n* = 5) and IL-22^−/−^ (*n* = 5) C57BL/6 mice were intratracheally (i.t.) infected with 10^4^ R265 yeast. Animals had their lungs surgically collected after euthanasia at 8 dpi, 13 dpi, and 18 dpi, macerated and cell suspension acquired. Single cell suspensions were stained for cytometry analysis. (A) Gating strategy representation. (B) Cell infiltration pattern of each group in lung histopathological slides stained with hematoxylin and eosin (H&E) ×3 magnification (scale of 500 μm). (C–F) CD45^+^Live^+^ (Leukocytes), (G–J) CD45^+^Live^+^CD3^+^ (T lymphocytes). (K–N) CD45^+^Live^+^F4/80^+^CD11b^+^SiglecF^−^ (infiltrating macrophages) (IM), (O–R) CD45^+^Live^+^F4/80^−^CD11b^+^Ly6G-SiglecF^+^ (eosinophils), (S–V) CD45^+^Live^+^F4/80^−^CD11b^+^Ly6G^+^ (neutrophils). ∗∗∗∗*p* < 0.0001, ∗∗∗*p* < 0.001, ∗∗*p* < 0.01, ∗*p* < 0.05 for comparisons between groups. (C, G, K, O, and S) (blue ) IL-23^−/−^ and (red , , , ) IL-22^−/−^ indicate comparison with WT. Representative data of 2 independent experiments. Data are represented as mean ± SD. (See also Figures S3 and S4).

Because differential fungal burden in knockout mice alters early-stage lung damage and cytokine profiles. A cytometry analysis was executed to assess the relative cell composition in the lungs during the infection (Figure 3A). Interestingly after evaluating the IL-22 source contribution in the lung microenvironment (Figure S3), we described that in the present mice model, the major source of IL-22 during the infection are γδ T cells and non-lymphoid cells (Figures S4D and S4H). Differently, when the infection is not undergone, non-leukocyte cells, or pneumocytes are the major source of IL-22 in the lung of uninfected animals, a scenario that is absent in the infection in course (Figure S4B). Generally, cell infiltration was compartmentalized in WT mice, and while IL-23^−/−^ mice displayed lesser lung cell infiltration, IL-22^−/−^ mice exhibited intensive and tissue diffuse cell infiltration at 18 dpi (Figure 3B). Although, the relative percentage of total leukocytes decreases through infection, the IL-22^−/−^ mice kept a lesser pronounced drop in their frequencies over time (Figures 3C and 3D). Nevertheless, T lymphocyte frequencies were lower at early stages of infection in both knockout mice, suggesting a reduced adaptative immunity capacity in terms of cell population in the lungs. However, only in the absence of IL-22 the frequencies of infiltrating macrophages and neutrophils kept higher late in the infection (Figures 3K–3N and 3S–3V), suggesting more innate immune response in terms of cell frequency in the lungs. Nevertheless, only the IL-22 absence impaired eosinophils frequency in the lungs during all stages of the infection (Figures 3O–3R). Therefore, the absence of IL-22 sustains the infiltrating inflammatory cell population during the infection, avoiding lung eosinophilia.

### The absence of IL-23 increases titan cell formation and capsular polysaccharide deposition in the lungs

**Figure 4:**
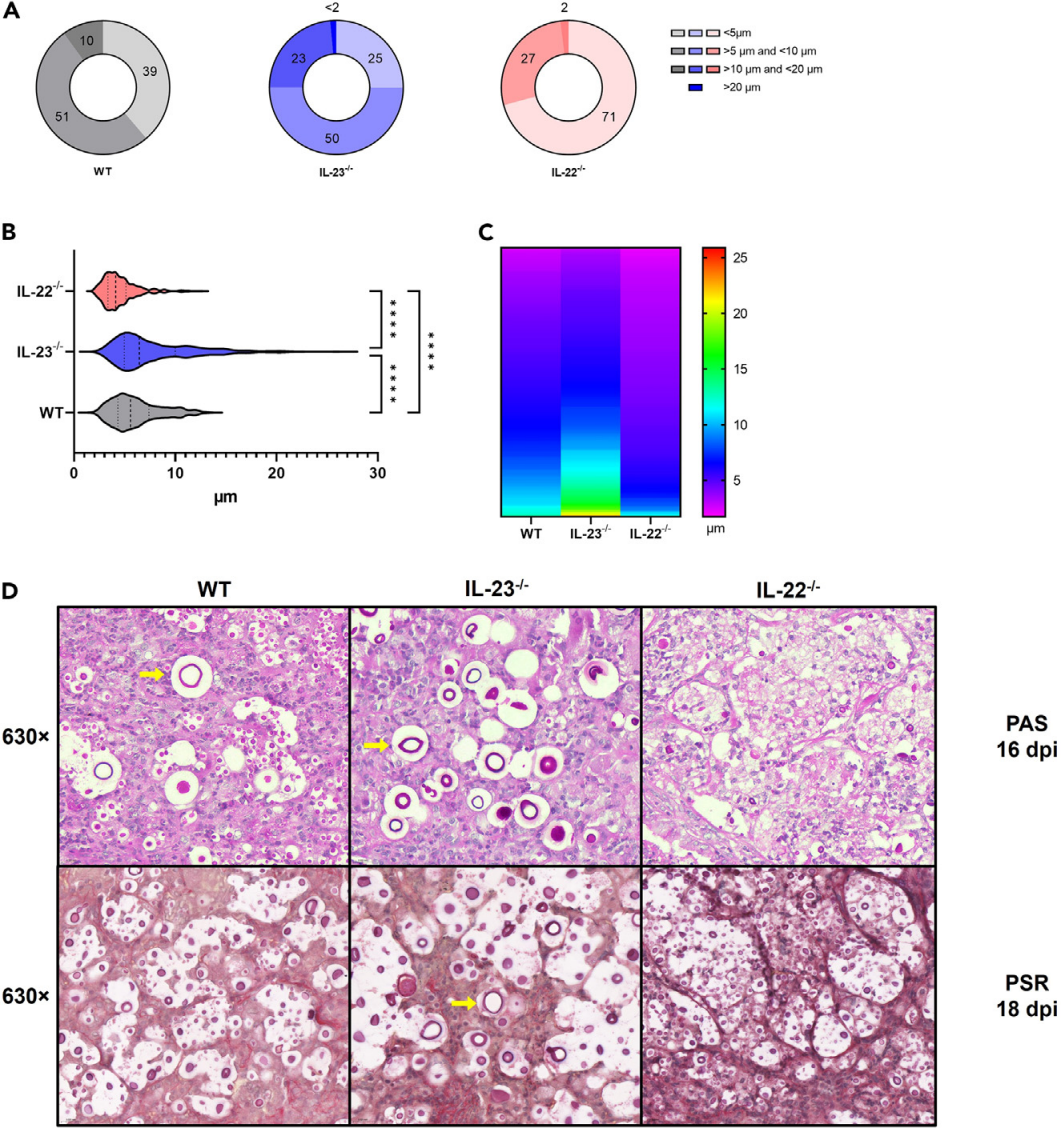
Analysis of fungal size and titanization wild type (WT) (*n* = 5), IL-23^−/−^ (*n* = 5), and IL-22^−/−^ (*n* = 5) C57BL/6 mice were intratracheally (i.t.) infected with 10^4^ R265 yeast and observed over time. (A–C) Animals had their lungs surgically collected after euthanasia at 18 dpi and taken for Picrosirius Red (PSR) staining. The analysis of the slides was performed using light microscopy on a scanning microscope (3DHISTECH Pannoramic MIDI Digital Slide Scanner). Cell size measurement was performed using the program (3DHISTECH Slide Viewer). Yeasts size was measured by diameter in 3 separated groups: under 5 μm (<5 μm), usual size between (>5 μm and <10 μm), usual titan cells between (>10 μm and <20 μm) and large titan cells above 20 μm (<20 μm); (*n* = 400) for all groups. (A) Donut chart with the relative percentage of yeasts by size for each group. (B) Box chart comparing relative diameter size between groups and (C) Heatmap showing diameter diversity between groups. (D) Representative images for each group of titan yeasts and normal yeasts on lung tissue at (16 dpi) using PAS staining and (18 dpi) using PSR staining (×630 of magnification). Photographs were taken using Motic EasyScan equipment for the 16 dpi time point. () yellow arrows indicate titan cells. ∗∗∗∗*p* < 0.0001 for comparisons between groups. Representative data of 3 independent experiments.

Titan cell formation is a virulence mechanism employed by cryptococci during infection.^40^ It is related to an uncontrolled immune response to the fungal pathogen, wherein the enlarged cell body hinders phagocytosis and causes damage to the lung tissue.^41^ Therefore, we measure the diameter of the yeasts in the lung tissue to identify the titan cells at 18 dpi. Those cells are above 10 μm in diameter with some of them reaching over 20 μm.^42^ Interestingly, around 50% of the yeasts are between 5 μm and 10 μm in diameter, considerably normal size, with 10% of titan cells in WT mice^43^ (Figure 4A). Remarkably, IL-23^−/−^ mice presented 23% of titan cells below 20 μm and less than 2% of titan cells above 20 μm, with the general yeasts size significantly higher than the WT group (Figures 4A, 4B, and 4D). In a different manner, IL-22^−/−^ mice presented 2% of titan cells, only below 20 μm and distinctly showcased 71% of yeasts below the normal size, less than 5 μm (Figure 4A), as illustrated by the major abundance of pinkish color in the heatmap chart (Figure 4C).

**Figure 5:**
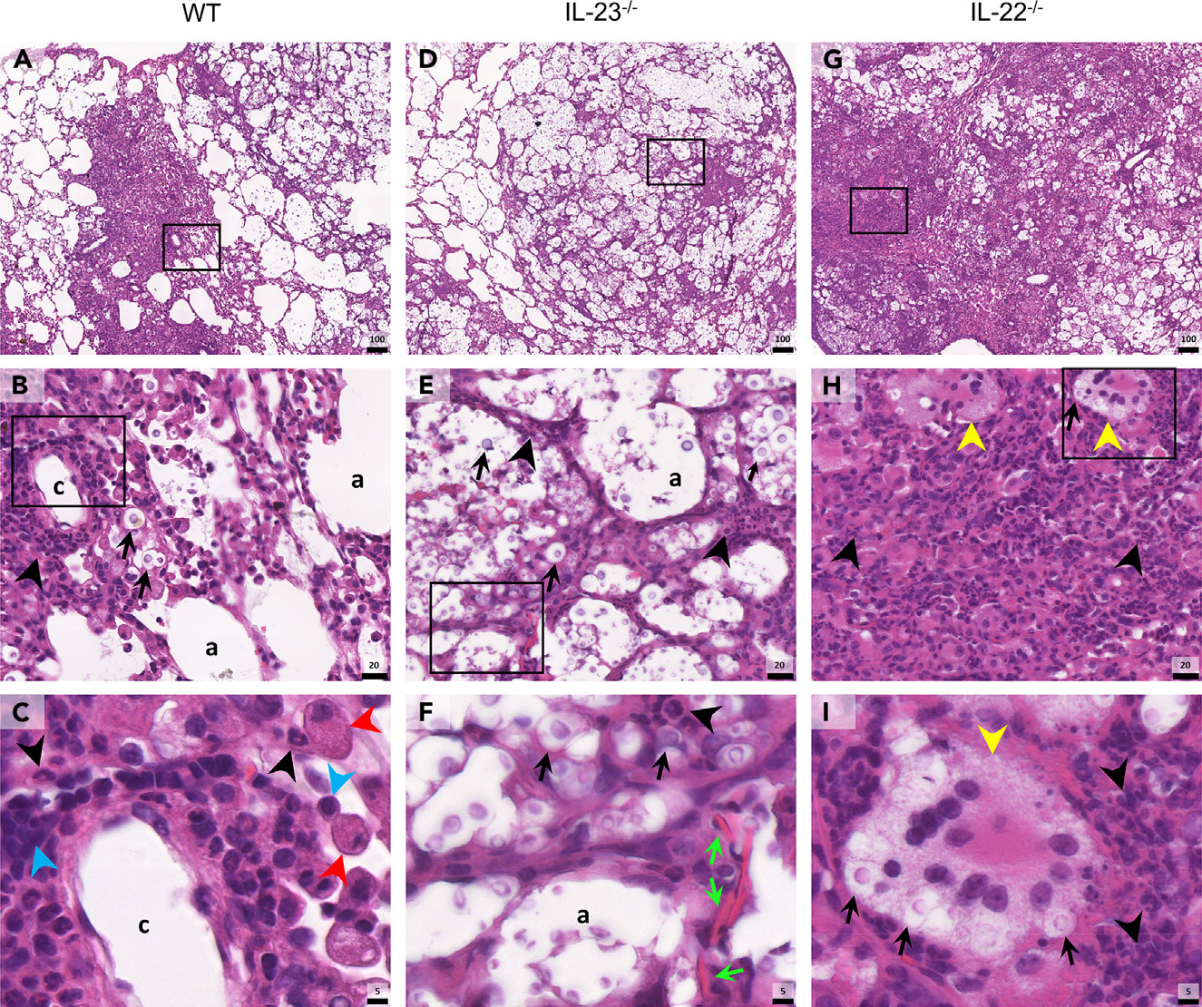
H&E staining of lung tissue of mice experimentally infected with R265 (A–C) Wild-type (WT) (*n* = 5), (D–F) IL-23^−/−^ (*n* = 5), and (G–I) IL-22^−/−^ (*n* = 5) C57BL/6 mice were intratracheally (i.t.) infected with 10^4^ R265 yeasts. Animals had their lungs surgically collected after euthanasia at 18 dpi and taken for hematoxylin and eosin (H&E) staining, (A, D, and G) ×10 magnification (scale of 100 μm), (B, E, and H) ×63 magnification (scale of 20 μm), (C, F, and I) ×200 magnification (scale of 5 μm). Black squares indicate magnification area, (c) indicate lung capillary, (a) indicate alveoli space, (↑) black arrows indicate R265 yeasts, () green arrows indicate Charcot-Leyden crystals, (▲) black arrowheads indicate infiltrating neutrophils, () yellow arrowheads indicate multinucleated giant cells (MGCs), () red arrowheads indicate macrophages, () blue arrowheads indicate lymphocytes. Representative images were taken with PANNORAMIC Midi II digital slide scanner. (See also Figures S5 and S6).

A unique feature of *Cryptococcus* spp. is their ability to produce a polysaccharide capsule during infection, which serves as a key virulence factor.^44^ This capsule plays a pivotal role in modulating the anticryptococcal immunity, ultimately leading to heightened fungal dissemination and compromised disease control.^14^ Thus, we manage to identify the capsular polysaccharide by a modified Alcian blue (MAB) staining method in lung histopathology analysis. Infected WT mice displayed more contained regions of R265 yeasts and capsular polysaccharides, limited mainly to alveolar areas surrounded by cellular infiltration (Figures 5A–5C). Differently, the IL-23^−/−^ mice exhibited large well-defined areas filled with yeasts (Figure 5D), they presented diversity in size with also the presence of titan cells (Figures 7E and 7F). Remarkably, the lung parenchyma of IL-22^−/−^ mice showed scarce less-defined regions of yeasts and capsular content (Figure 5G). Interestingly, they presented the capsular polysaccharide content inside phagocytes, mainly in MGCs with multiple engulfed yeasts (Figures 5H and 5I). Therefore, IL-23^−/−^ seems to present histopathological features related to an uncontrolled lung fungal burden, different from the IL-22^−/−^ that shows features more consistent with a more competent anticryptococcal immunity.

### IL-22 deficiency enhances pulmonary edema, fibrosis, and intensifies immune cell recruitment leading to excessive lung damage

**Figure 6:**
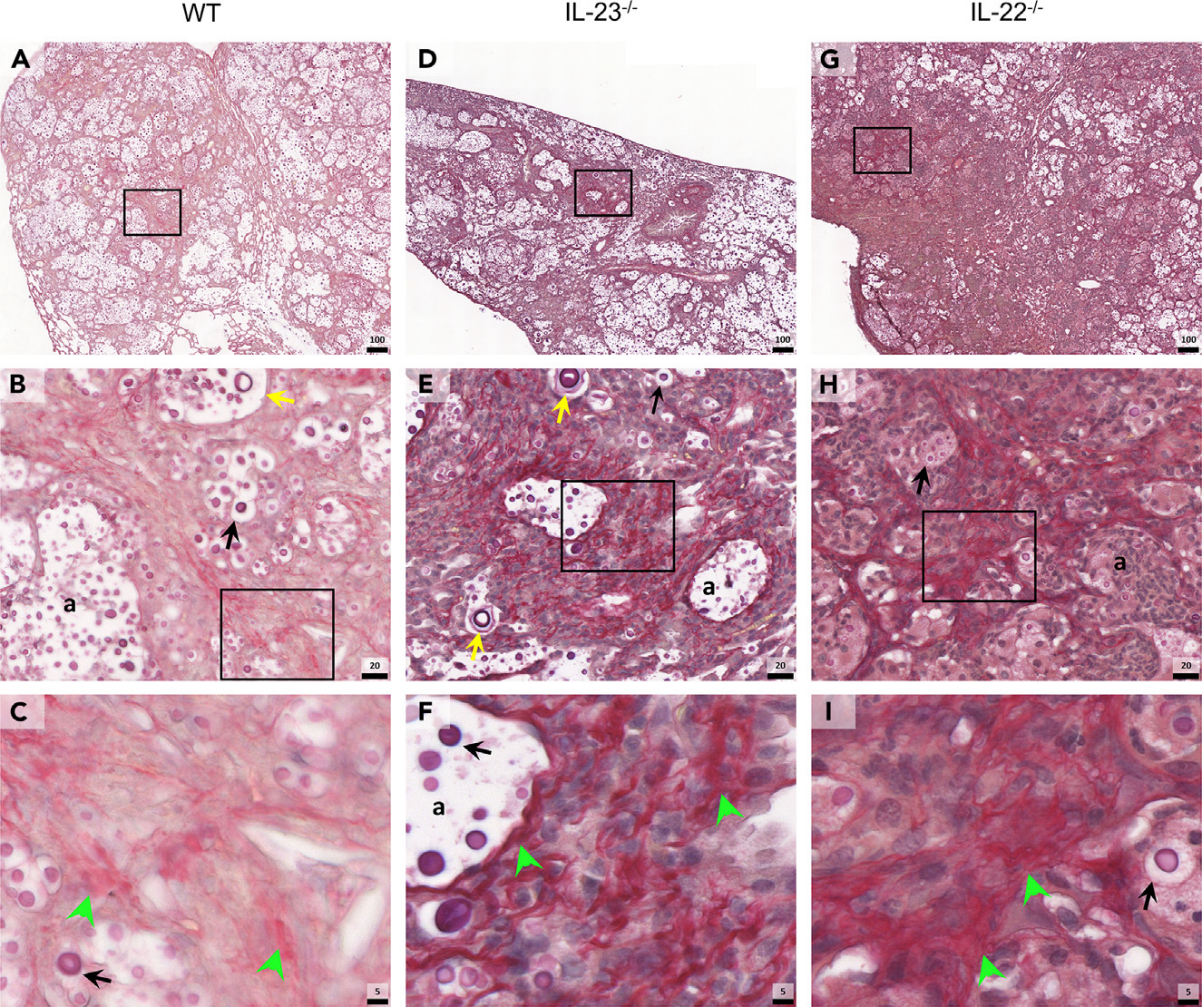
Picrosirius red staining of lung tissue of mice experimentally infected with R265 (A–C) Wild-type (WT) (*n* = 5), (D–F) IL-23^−/−^ (*n* = 5) and (G–I) IL-22^−/−^ (*n* = 5) C57BL/6 mice were intratracheally (i.t.) infected with 10^4^ R265 yeasts. Animals had their lungs surgically collected after euthanasia at 18 dpi and taken for PSR, (A, D, and G) ×10 magnification (scale of 100 μm), (B, E, and H) ×63 magnification (scale of 20 μm), (C, F, and I) ×200 magnification (scale of 5 μm). Black squares indicate magnification area, (a) indicate alveoli space, (↑) black arrows indicate R265 yeasts, () yellow arrows indicate titan cells, () green arrowheads indicate collagen fibers. Representative images were taken with PANNORAMIC Midi II digital slide scanner. (See also Figure S7).

Pulmonary cryptococcosis impacts dramatically in the lung parenchyma, leading to lung tissue damage and pneumonia.^45^^,^^46^ Therefore, we evaluated the general morphology of the lungs by histopathology to access the lung integrity in R265 infected mice 18 dpi. Although WT mice presented perivascular cell infiltration mainly composed by neutrophils and macrophages, (Figures 6A–6C), IL-22^−/−^ mice exhibited intense neutrophil infiltration and multinucleated giant cells (MGCs) with engulfed cryptococci (Figures 6G–6I). The lungs of IL-23^−/−^ mice presented scarce regions of infiltrating cells and a higher number of yeasts (Figures 6D–6F). Particularly, IL-23^−/−^ mice displayed Charcot-Leyden crystals in the lung tissue (Figure 6F), they are commonly formed by eosinophil-derived proteins, and are associated with eosinophilic inflammation.^47^

**Figure 7:**
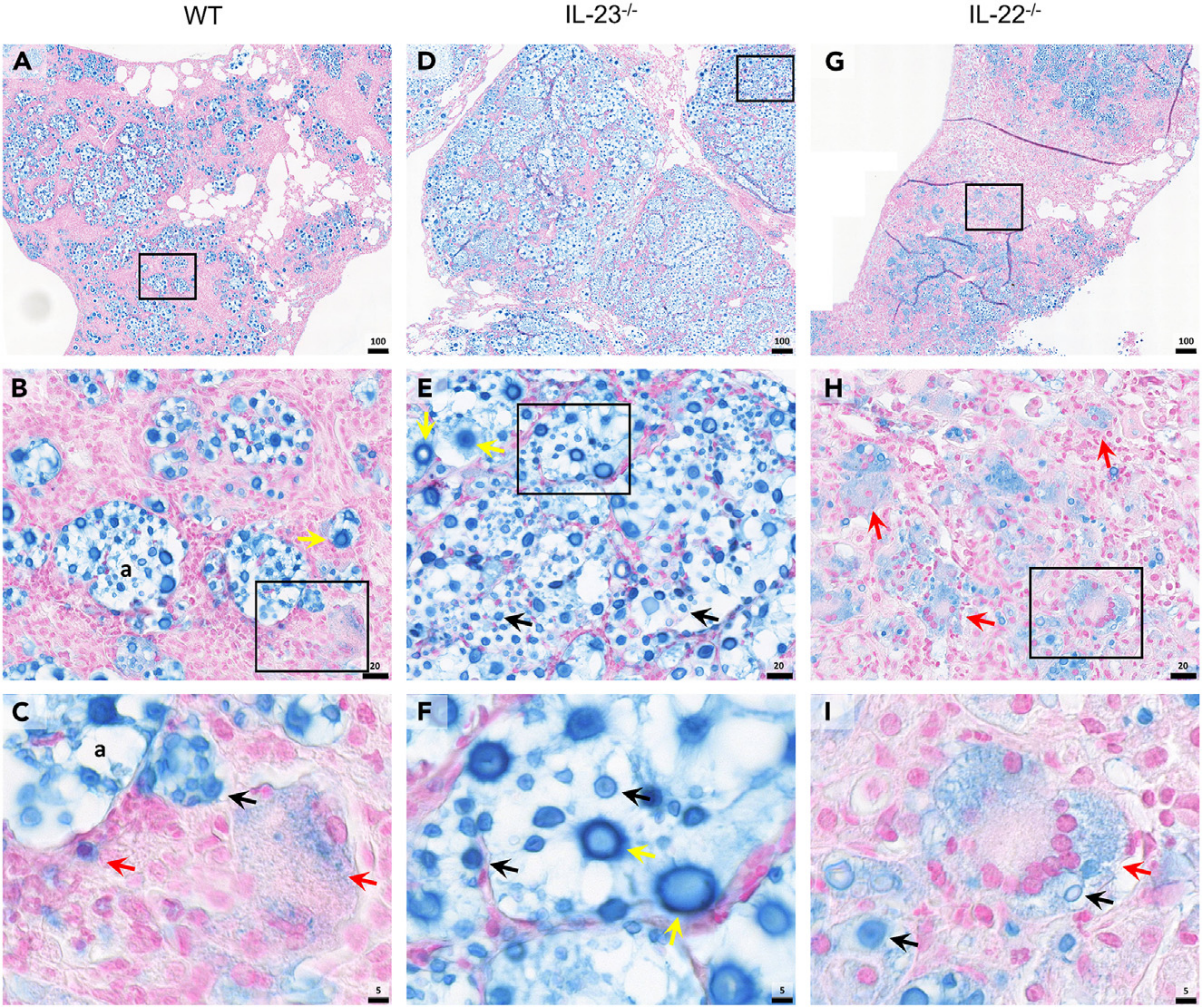
Modified Alcian blue staining of lung tissue of mice experimentally infected with R265 (A–C) Wild-type (WT) (*n* = 5), (D–F) IL-23^−/−^ (*n* = 5), and (G-I) IL-22^−/−^ (*n* = 5) C57BL/6 mice were intratracheally (i.t.) infected with 10^4^ R265 yeasts. Animals had their lungs surgically collected after euthanasia at 18 dpi and taken for modified Alcian blue (MAB) staining, (A, D, and G) ×10 magnification (scale of 100 μm), (B, E, and H) ×63 magnification (scale of 20 μm), (C, F, and I) ×200 magnification (scale of 5 μm). Black squares indicate magnification area, (a) indicate alveoli space, (↑) black arrows indicate R265 yeasts, () red arrows indicate capsular polysaccharides inside phagocytes, () yellow arrows indicate titan cells. Representative images were taken with PANNORAMIC Midi II digital slide scanner.

The tissue damage originated by the inflammatory response triggers lung fibrosis and collagen deposition, exacerbating the loss of lung function, and hindering regenerative responses.^48^^,^^49^ Therefore, we evaluated the lung collagen deposition in the infected lung tissue, and both IL-23 and IL-22 absence resulted in a more intense lung fibrosis compared to the WT group (Figure 7). WT displayed scarce regions of collagen fiber deposition identified with the picrosirius red staining (PSR) (Figures 7A–7C) and Masson’s trichrome (MT) staining (Figure S5A). On the contrary, IL-23^−/−^ mice presented more developed and organized collagen fibers across the lung parenchyma (Figures 7D–7F), and the IL-22^−/−^ mice displayed entangled collagen fiber deposition between the intense cell infiltration (Figures 7G–7I). Yeast can also be identified by this staining method, allowing to see the differences in size and identification of titan cells (Figures 7B and 7E).

We also evaluated the diffuse alveolar damage (DAD) score that consider many parameters such as alveolar collapse, alveolar inflammation, and alveolar edema in extension and intensity within the lung parenchyma (Table 1; Figure S4C). It was observed intense damage to the lung parenchyma of IL-22^−/−^ animals at 16 dpi, accompanied by a large amount of mucus in the airways and collagen deposition (Table 1; Figures S5A and S5B). The lungs of IL-22^−/−^ animals, unlike the other groups, were filled with cellular infiltrates, characterized by intense neutrophilia (Figure S6). In accordance with the lung damage observed in the knockout animals, it was also noted that both deficient groups had elevated basal lung resistance when the lung resistance assay was performed under controlled mechanical ventilation (Figure S7A). Due to excessive mucus production, IL-22^−/−^ animals exhibited low responsiveness in the lung resistance assay with increasing doses of methacholine, as the obstructed airways limited the nebulization of the drug (Figure S7B).

**Table 1:** Lung histopathological score of *C. deuterogattii* infected animals

Parameter	Score range	SHAM (*n* = 5)	WT (*n* = 4)	IL-23^−/−^(*n* = 4)	IL-22^−/−^(*n* = 5)
Alveolar collapse	0–16	0 ± 0	6.44 ± 0.62	5.54 ± 0.72	15.38 ± 0.76
Alveolar inflammation	0–16	0 ± 0	6.59 ± 0.9	5.11 ± 0.61	16.00 ± 0
Alveolar Edema	0–16	0 ± 0	4.65 ± 0.82	3.86 ± 1.32	15.68 ± 0.39
DAD	0–48	0 ± 0	17.68 ± 0.49	14.51 ± 1.46	47.06 ± 1.16
Peribronchial cell infiltration	0–4	0 ± 0	3.60 ± 0.38	3.48 ± 0.38	4.00 ± 0
Lung collagen^a^	0–100	17.14 ± 2.38	26.15 ± 1.09	21.19 ± 1.09	32.34 ± 2.43
Bronchial mucus^a^	0–100	1.68 ± 0.24	23.82 ± 4.15	24.01 ± 4.15	44.90 ± 4.33

a Both parameters are expressed in percentage.

## Discussion

*Cryptococcus deuterogattii* (R265) together with other *Cryptococcus* species, has emerged as one of the most significant concerning fungal pathogen.^15^ This implies that a need for deeper comprehension of the immunological aspects of the disease is decisive to address and manage cryptococcosis clinically. In this work we show unique features of immune activation during R265 infection in mice. Nevertheless, it is evident that the lung microenvironment seems to be decisive for disease control and restrictive for systemic fungal dissemination, therefore we showcased a mechanism in which the IL-22 cytokine plays a pivotal role in regulating and limiting excessive inflammatory lung responses. In the absence of the IL-22 cytokine, the mice survival rate dropped significantly, and this is mainly relatable with the excessive inflammatory cytokines such as IFN-γ and IL-17A being produced in the lungs. Indeed, the IFN-γ and IL-17A production in the lung site, induces an extensive myeloid cell infiltration, such as neutrophils and macrophages in response to adaptative local responses by resident cells.^50^ As a result, the robust production of IFN-γ and IL-17A was responsible for the intense recruitment of cells in the absence of IL-22. Significantly, the absence of IL-23 exclusively led to the production of IFN-γ without IL-17A, highlighting the crucial role of IL-17A in facilitating increased cell infiltration in the lung. Furthermore, the absence of the IL-22 cytokine exacerbates lung inflammation, resulting in significantly elevated levels of IL-1β, TNF-α, and IL-12 during infection. This increase is linked to antifungal immunity, as TNF-α and IL-12 are essential for amplifying a protective Th1 cell-mediated response against pulmonary cryptococcosis.^51^ Notably, IL-5 and IL-4 levels were also reduced in these mice, suggesting enhanced resistance to infection.^52^ IL-22, on the other hand, emerges as a regulatory factor in modulating this phenomenon specifically within the context of R265 infection. Notably, IL-22 production is not limited solely to activated T lymphocytes; thus, even in the absence of the IL-23 axis and IL-17A production, IL-22 could be generated by non-lymphoid sources.^53^ Hence, IL-22 is vital for survival, as IL-22-deficient mice perished earlier than IL-23-deficient mice. Moreover, during the initial phase of R265 infection, a reduced lung T cell ratio was observed in the absence of either IL-22 or IL-23 alone. This observation indicates a compromised adaptive response within the pulmonary microenvironment, particularly regarding T cell recruitment, despite an amplified production of IFN-γ in both knockout groups. Thus, the augmentation of the Th1 profile activation demonstrates a regulatory property of this axis dependent on IL-22 production. However, the primary source of IL-22-producing cells during the infection was predominantly Tγδ lymphocytes and non-lymphoid cells. Therefore, despite the reduction in conventional T lymphocytes over the course of the infection, in non-IL-22-deficient animals, the production of this cytokine can be sustained by these populations throughout the infection. Nevertheless, cryptococcosis itself is known for leading progressive lymphopenia in competent mice^54^ and can overcome the immune response in T cell suppressed individuals, such is the case for patients with low TCD4 counts during acquired immunodeficiency syndrome (AIDS), displaying higher susceptibility for *Cryptococcus* spp. infection.^55^ Concurrently, the production of IFN-γ and IL-17A production is correlated with a Th1/Th17 axis activation in the infected lung microenvironment, and this represents a competent anticryptococcal response.^56^ However, the excess activation of this axis tends to lead to an uncontrolled inflammatory and less healing response, which is critical during lung excessive damage and cell infiltration.^57^ This scenario causes less regulated immune response against infection and exacerbates the clinical manifestation of lung pneumonia.^58^

Although, more clinical manifestations of inflammatory responses happened earlier in the absence of IL-22, and in the absence of IL-23 cytokine as well. Nonetheless, despite the IL-22 production could be originated from activated epithelial cells source,^59^ its production can be achieved together with IL-17A by IL-23 stimulated T lymphocytes.^60^ This mechanism is triggered by the antifungal immunity, mainly during Th17 cell activation through stimulated phagocytes in the context of cryptococcosis.^61^^,^^62^ Therefore, IL-23 also plays a pivotal role in the anticryptococcal immunity, as its administration also confers protection during mice *C. neoformans* infection.^28^ Simultaneously, in the context of R265 infection, the production of IL-23 must be intricately to infection control for similar reasons. In its absence, there is a heightened occurrence of lung damage, accompanied by a substantial increase in the systemic spread of fungal burden. Curiously, titan cell formation happened intensively more in the absence of IL-23, sustaining the case of poor disease control. Nevertheless, titan cell formation is also relatable with higher fungal dissemination to the central nervous system during R265 infection,^46^ as titan yeast formation is linked to a higher lung fungemia.^63^ Therefore, more fungal burden and more probability for the fungus to reach farther systemic areas. The heightened production of Th2-type cytokines, such as IL-4, IL-5, and IL-33, in IL-23-deficient animals may be closely associated with the increased presence of titan cells. This connection arises from the uncontrolled infection driving these morphological changes, which are linked to *Cryptococcus* resistance. Furthermore, eosinophilia plays a role in this mechanism, yet it was significantly reduced during infection in IL-22-deficient animals.^64^ Conversely, during *C. neoformans* infection, titan cell formation is linked to less phagocytosis and less phagocytes transporting cells to farther areas by the trojan-horse mechanism,^65^ which consists in phagocytes carrying out the yeast inside through the tissue barriers of the host, such as the central nervous system.^32^ Therefore, during R265 infection the higher systemic dissemination is possibly linked to the higher lung fungal burden and tissue destruction, as this infection tends to lead in death by pneumonia and not by meningoencephalitis.^8^ Moreover, the R265 infection *in vitro* induces much more IL-22 production than other pathological species of *Cryptococcus* genera.^66^ Our study demonstrates, using an experimental mice model that the *in vivo* infection caused by R265 indeed results in a significant accumulation of IL-22 in both the lungs and brain of infected animals. Therefore, because IL-22 is produced in response to extensive epithelial activation and issue damage for achieving repair, is expected that the R265 lung infection causes a positive feedback response in attempt to avoid excessive lung damage.

Undoubtedly, in the absence of IL-22, lung damage was completely heighted as well the inflammatory response. In fact, only the experimental animal group without IL-22 cytokine displayed extensive and increasingly fungemia throughout the entire infection course, despite the fungal burden control in the lungs. Henceforth, IL-22 seems to have a pivotal role in controlling the airway-blood barrier, as in its absence, extensive cell infiltration in the lungs and poor fungal containment was observed. Accordingly, IL-22 functions, comprise not only for healing and tissue repair of epithelial barriers,^25^ but also for inducing β-defensin production in the alveoli spaces during cryptococcosis control,^22^ also improving tight junction formation and decreasing inflammatory responses.^67^ *Cryptococcus* spp. dissemination is also dependent on the capability to overcome the host systemic barriers, for that, the fungus uses molecules present on its capsular content to facilitate adhesion, and digest cell tight junction proteins.^68^^,^^69^ Specifically, the hyaluronic acid produced by cryptococci, is a ligand for the CD44 molecule, and this interaction facilitates the yeast adhesion to the epithelial barriers,^70^ allowing close yeast-cell interaction to engage transport mechanisms such paracellular, transcellular and trojan-horse within phagocytes.^37^ Furthermore, the urease produced by the fungus digests the cell tight junctions granting easy paracellular passage through the barriers.^71^ For this reason, the deprivation of a regulatory IL-22 function on the lung and brain barriers could lead to a worsened fungal containment, as the epithelial excessive activation engages CD44 expression.^5^^,^^72^ In this case, the unfavorable tight junction engagement by IL-22 absence could intensify the paracellular fungal passage. Interestingly, as opposed to the IL-23 deficiency, the lack of IL-22 led to more than 70% of yeasts under the expected size, this also could explain why a consistent higher fungemia of observed, as less sized yeasts are easier be phagocytosed and to escape the lung barrier. Furthermore, the reduction of normal sized yeasts is also explained by the intense inflammatory response in the absence of IL-22 production, as an extensive macrophage and neutrophil infiltration was limiting fungal proliferation inside the lungs. Interestingly, IL-22 deficient animals also show reduced IL-33 production in the lungs, which may lead to decreased Th2 axis activation during cryptococcosis and a lower presence of allergic-type responses. This contrasts with IL-23 deficient animals, which tend to exhibit a stronger Th2 profile and greater pulmonary resistance during infection.

A remarkable mechanism of R265 is the Th1 profile downregulation during the infection, surpassing even the activation of this immune response of other cryptococcal species in co-infection experiments.^34^ This mechanism is associated with an enhanced Th2 response, characterized by less disease control and an intense eosinophilia.^64^ This in turn, contributes to heightened allergic inflammation exacerbating the overall lung damaging processes.^73^ Moreover, the IL-22 role in this process remains incompletely understood. Nevertheless, a substantial portion of the IL-22 function is intricately linked with the regulation of the epithelial surfaces. Thus, the interplay between the immune lung microenvironment and the impact into the lung barriers during the cryptococcal infection emerges as a crucial point of convergence for the IL-22 role. Accordingly, IL-22 regulates neutrophilic infiltration in the lungs, thereby, restricting the effectiveness of neutrophilic proteases on the lung microenvironment, limiting the lung damage in case of intracellular infection.^74^^,^^75^ Therefore, in our work, we show that the lack of IL-22 facilitates not only lung neutrophilia but also the increase in macrophage infiltration, showcasing intense alveolar cellularity, and multinucleated giant cells (MGCs) with extended lung damage. Especially, MGCs are linked to an intense inflammatory response and intracellular cryptococci confinement,^76^ which it reinforces the important participation of IL-22 in controlling excessive inflammatory responses. It elucidates the reason behind the premature mortality of knockout mice lacking IL-22 production, attributing it to heightened inflammation in crucial organs, particularly the lungs and brain. This heightened inflammatory response ultimately culminated in the deterioration of lung tissues, resulting in the untimely demise of these mice.

Fascinatingly, the MGCs were replete intracellularly with the capsular polysaccharides of R265. These molecules primarily constituted of glucuronoxylomannan (GXM) and, to a lesser extent of glucuronoxylomannogalactan (GXMGal),^2^ showcase immunomodulatory characteristics, predominantly oriented toward pro-apoptotic mechanisms and the downregulation of cell activation.^77^ Therefore, its presence in the lung microenvironment can deprive anticryptococcal immune activation.^78^ However, even in the absence of IL-22, which tends to reduce the severe burden of R265 in the lungs, there is a notable presence of MGCs filled with polysaccharides in the lung parenchyma. This might contribute to a diminished ability of those phagocytes to effectively clear engulfed yeasts, even in the face of substantial cell infiltration. Consequently, IL-22 appears crucial for containing yeast in the alveoli and preventing the widespread distribution of these capsular contents throughout the lung tissue.

Notably, eosinophilia was disfavored in the absence of IL-22, a phenomenon that was associated with less IL-13 production. Therefore, a restrained Th2 response ensued, leading to a more prevalent Th1/Th17 response. On contrary, the IL-23 absence led to an intense fungal burden with more IL-13 production and sustained eosinophil ratio in the lungs. In this sense, Charcot-Leyden crystals were also present in the lung parenchyma of those infected animals. They are composed by the crystallization of protein content within the granules of eosinophils, and their presence is closely linked to an intense allergic activation and eosinophil abundance in the affected tissue.^79^ Consequently, the absence of the IL-23 cytokine leads to a restraint in the production of IL-17A.^80^ Furthermore, during *C. neoformans* infection, IL-23 production seems to reduce eosinophilia and the allergic lung.^27^ Similarly, our data also reinforces these mechanisms during the R265 lung infection. Therefore, the integrity of the airway-blood barrier in the context of R265-induced eosinophilic allergy also relies on the IL-22 production as well as the promotion of Th2 profile, characteristic of R265 infection.^81^

In summary, our findings provide crucial insights into the meticulous regulation of anticryptococcal immunity during R265 infection by the IL-22 cytokine, shedding light on its implications for the overall disease outcome. Therefore, we showcase a role of the IL-22 cytokine as a key factor for the retainment of R265 infection in the lungs and in limiting intensive lung inflammation during R265 cryptococcosis. The pivotal role of IL-22 in the containment of cryptococci and its effectiveness in preventing lung damage, underscore the critical importance of immune regulation during fungal lung infections, and emphasizes the role of IL-22 regulatory properties during this infection. Interestingly, IL-22 plays a role in maintaining the Th2 profile during R265 infection. Additionally, the integrity of the systemic barriers proves essential for lung containment. Nevertheless, during immunosuppression, mechanisms safeguarding barrier integrity may be compromised, thereby facilitating systemic dissemination. Therefore, because IL-22 cytokine significantly seems to limit fungal systemic dissemination but also helps in maintaining R265 in immunopathological features, it becomes imperative to explore its role not only in opportunistic infections but also in primary and more aggressive infections, such as those caused by the R265 species. This endeavor is essential in gaining a more profound insight into the disease and elucidating the strategies necessary for upholding the overall lung homeostasis during the aggressive R265 infection.

### Limitations of the study

The present study analyzed pulmonary immunophysiology during experimental infection in mice. Although the progression of infection in humans differs from that observed in mice, the virulence factors of the fungus differentially modulate the components of the immune system in human cells and other mammals.^82^ Thus, despite indications of the role of IL-22 in the immunomodulation of *C. deuterogattii* infection in mice, further studies are needed to more clearly elucidate the immunopathological impacts of this infection in human hosts.

## Resource availability

### Lead contact

Further information and requests for resources and reagents should be directed to and will be fulfilled by the lead contact, Celio Geraldo Freire-de-Lima (celio@biof.ufrj.br).

### Materials availability

This study did not generate new unique reagents.

### Data and code availability

• Date: This paper does not report original data.
• Code: This paper does not report original code.
• All other requests: Any additional information required to reanalyze the data reported will be shared by the lead contact upon request.

## Acknowledgments

We would like to extend a special thanks to the undergraduate research students from the Immunomodulation Laboratory (LABIM-IBCCF-UFRJ) Jesica Mel da Silva Faria and Gustavo José Makhoul de Almeida who dedicated their time and energy to the completion of this work. We are grateful to the Instituto de Biofísica Carlos Chagas Filho of the Universidade Federal do Rio de Janeiro (IBCCF-UFRJ), the Instituto de Microbiologia Paulo de Góes (IMPG-UFRJ), the Laboratório de Animais Transgênicos (LAT-IBCCF-UFRJ), the IMPG-UFRJ Flow Cytometry Core Facility and the Plataforma de Imunoanálise (PIA-IBCCF-UFRJ) for providing infrastructure for animal housing during experimentation and structural support for the analysis platforms. We also extend our thanks to the Laboratório de Glicobiologia (LABGLICO-IBCCF-UFRJ), the Laboratório de Imunobiotecnologia (LIBTEC-IMPG-UFRJ), the Laboratório de Investigação Pulmonar (LIP-IBCCF-UFRJ), the Laboratório de Imunologia Molecular e Celular (LIMC-IBCCF-UFRJ), the Laboratório de Imunofisiologia (LIF-IBCCF-UFRJ), and the Laboratório de Glicobiologia Estrutural e Funcional (LAGEF-IBCCF-UFRJ) for their support in recommending, providing, and utilizing reagents.

Funding sources: this work was supported by the 10.13039/501100003593Conselho Nacional de Desenvolvimento Científico e Tecnológico (CNPq), the 10.13039/501100004586Fundação Carlos Chagas Filho de Amparo à Pesquisa do Estado do Rio de Janeiro (FAPERJ), the Coordenação de Aperfeiçoamento de Pessoal de Nível Superior (CAPES) and the Fundação Oswaldo Cruz (FIOCRUZ).

## Author contributions

Conceptualization, I.D.-L., D.D.-R., H.L.d.M.G. and C.G.F.-d.-L.; methodology, I.D.-L., H.L.d.M.G., and C.G.F.-d.-L.; formal analysis, I.D.-L.; investigation, I.D.-L., A.G., M.M., J.C.G.-d.-O., I.M.F.-d.-S., and E.B.d.S.-J.; resources, A.M., D.O.N., F.F.C., L.F.-d.-L., and L.D.B.-G.; writing—original draft, I.D.-L.; writing—review and editing, I.D.-L., H.L.d.M.G., and C.G.F.-d.-L.; supervision, D.D.-R., H.L.d.M.G., and C.G.F.-d.-L.; project administration, H.L.d.M.G. and C.G.F.-d.-L.; funding acquisition, H.L.d.M.G. and C.G.F.-d.-L.

## Declaration of interests

The authors declare no competing interests.

## STAR★Methods

### Key resources table

**Table:** 

REAGENT or RESOURCE	SOURCE	IDENTIFIER
**Antibodies**		

BD Pharmingen™ Purified Rat Anti-Mouse CD16/CD32 (Mouse BD Fc Block™)	BD©	Cat#553141; RRID: AB_394656
BD Horizon™ Fixable Viability Stain 510	BD©	Cat#564406; RRID: AB_2869572
BD Horizon™ BV421 Rat Anti-Mouse CD45	BD©	Cat#563890; RRID: AB_2651151
BD Pharmingen™ APC-Cy™7 Hamster Anti-Mouse CD3e	BD©	Cat#557596; RRID: AB_396759
BD Pharmingen™ APC Rat Anti-Mouse F4/80	BD©	Cat#566787;RRID: AB_2869866
BD Pharmingen™ PE-Cy™7 Rat Anti-CD11b	BD©	Cat#552850; RRID: AB_394491
BD Pharmingen™ FITC Rat Anti-Mouse Ly-6C	BD©	Cat#561085; RRID: AB_10584332
BD OptiBuild™ BV650 Rat Anti-Mouse Ly-6G	BD©	Cat#740554; RRID: AB_2740255
BD Pharmingen™ PE Rat Anti-Mouse Siglec-F	BD©	Cat#552126; RRID: AB_394341
BD Horizon™ BV786 Rat Anti-Mouse CD45	BD©	Cat#564225; RRID: AB_2716861
BD Horizon™ BV711 Rat Anti-Mouse CD19	BD©	Cat#563157; RRID: AB_2738035
BD Pharmingen™ PE-Cy™7 Hamster Anti-Mouse TCR β Chain	BD©	Cat#560729; RRID: AB_1937310
BD Pharmingen™ APC-Cy™7 Mouse Anti-Mouse NK-1.1	BD©	Cat#560618; RRID: AB_1727569
BD Pharmingen™ Alexa Fluor® 647 Mouse Anti-Mouse RORγt	BD©	Cat#562682; RRID: AB_2687546
Alexa Fluor® 488 anti-mouse TCR γ/δ Antibody	BioLegend©	Cat#118127; RRID: AB_2562770
PE anti-mouse IL-22 Antibody	BioLegend©	Cat#516404; RRID: AB_2124255

**Chemicals, peptides, and recombinant proteins**		

Ketamin 10%	Syntec©	N/A
Xylasin 2%	Syntec©	N/A
Pancuronium bromide	Sigma-Aldrich	Cat#P1918-10MG
Methacholine Chloride	Sigma-Aldrich	Cat#PHR1943-1G

**Critical commercial assays**		

Murine TNF-α Standard ABTS ELISA Development Kit	PeproTech©	Cat#900-K54
Murine IL-4 Standard TMB ELISA Development Kit	PeproTech©	Cat#900-T49
Murine IL-13 Standard ABTS ELISA Development Kit	PeproTech©	Cat#900-K207
Murine IL-22 Standard ABTS ELISA Development Kit	PeproTech©	Cat#900-K257
Murine IL-12 Standard ABTS ELISA Development Kit	PeproTech©	Cat#900-K97
Murine IL-6 Standard ABTS ELISA Development Kit	PeproTech©	Cat#900-K50
Murine IL-5 Standard ABTS ELISA Development Kit	PeproTech©	Cat#900-K406
Murine IL-10 Standard ABTS ELISA Development Kit	PeproTech©	Cat#900-K53
Murine IL-17A Standard ABTS ELISA Development Kit	PeproTech©	Cat#900-K382
Murine IFN-γ Standard ABTS ELISA Development Kit	PeproTech©	Cat#900-K98
DuoSet© ELISA DEVELOPMENT SYSTEM Mouse IL-1β/IL-1F2	R&D Systems	Cat#DY401
Human/Mouse TGF beta 1 Uncoated ELISA	Thermo Fisher Scientific	Cat#88-8350
Mouse IL-33 Uncoated ELISA	Thermo Fisher Scientific	Cat#88-7333
Mouse IL-23 Uncoated ELISA	Thermo Fisher Scientific	Cat#88-7230
Cell Activation Cocktail (with Brefeldin A)	BioLegend©	Cat#423303
BD Cytofix/Cytoperm™ Plus Fixation/Permeabilization Solution Kit with BD GolgiStop™	BD©	Cat#554715

**Experimental models: organisms/strains**		

*Cryptococcus deuterogattii*, serotype B, R265 hypervirulent species, molecular type VGII, MAT α	Diniz-Lima et al.^42^	N/A
Wild-type C57BL/6 mice	Diniz-Lima et al.^42^	N/A
IL-22 knockout C57BL/6 mice	This paper	N/A
IL-23 knockout C57BL/6 mice	This paper	N/A

**Software and algorithms**		

GraphPad Prism 9	Dotmatics©	https://www.graphpad.com/
3DHISTECH Slide Viewer	3DHISTECH Ltd©	https://www.3dhistech.com/
ImageJ	NIH	https://imagej.net/
FlowJo™ VX	BD©	https://www.flowjo.com/
Buxco Pulmonary Mechanics Processing System	Buxco Electronics, Wilmington, NC, USA	https://www.datasci.com/products/buxco-respiratory-products/pulmonary-function-testing

**Other**		

SpectraMax® M5 Multi-Mode Microplate Reader	Molecular Devices©	Cat#M5
Motic EasyScan Pro	Motic©	https://www.motic.com/As_MoticEasyScanPro/
3DHISTECH Pannoramic MIDI II Digital Slide Scanner	3DHISTECH Ltd©	https://www.3dhistech.com/research/pannoramic-digital-slide-scanners/pannoramic-midi/
BD LSRFortessa™ X-20 Cell Analyzer	BD©	https://www.bdbiosciences.com/en-be/products/instruments/flow-cytometers/research-cell-analyzers/bd-lsrfortessa-x-20
FinePoint R/C Buxcos Platform	Buxco Electronics, Sharon, CT, USA	https://www.datasci.com/products/software/finepointe-software

### Experimental model

#### *Cryptococcus deuterogattii* maintenance

*Cryptococcus deuterogattii*, serotype B, R265 hypervirulent species, molecular type VGII, MAT α. Was kept in an incubator at 30°C for 48 h in Sabouraud medium, followed by Minimum medium for 120 h, containing: 2.7 g/L of glucose (C_6_H_12_O_6_); 2.5 g/L of heptahydrated magnesium sulfate (MgSO_4_.7H_2_O); 4.0 g/L of monobasic potassium phosphate (KH_2_PO_4_); 10 g/L of glycine (C_2_H_5_NO_2_); 0.001 g/L of thiamine (C_12_H_17_N_4_OS) before each inoculation.

#### Animal selection

Isogenic male mice of the C57BL/6 strain, wild-type (WT), and knockouts IL-23^−/−^ and IL-22^−/−^ were selected. They were males, aged between 8 and 12 weeks, weighing an average of 20 g. All mice originated from the Animal Facility of the Laboratório de Imunomodulação (LABIM) at the Instituto de Biofísica Carlos Chagas Filho (IBCCF) of the Universidade Federal do Rio de Janeiro (UFRJ). The animals were kept in sterile cages, grouped, under standardized conditions at temperature of (22°C–23°C) and 12 h light and 12 h dark cycles, with commercial feed and drinking water provided *ad libitum* at the IBCCF animal facility. The mice were euthanized according to the approved criteria of the Ethics Committee on Animal Experimentation (CEUA) of UFRJ. The use of the animals in the present study was approved by the Ethics Committee on Animal Use (UFRJ) under number 092/21.

### Method details

#### Inoculation in the experimental infection

After culturing the fungus, 1 mL of the last pre-inoculum was centrifuged at 4985 × G for 3 min (Micro High Speed Refrigerated Centrifuge VS-15000 CFN II CE), resuspended in 50 mL of sterile saline solution with phosphate buffer (PBS) (NaH_2_PO_4_ 0.538 g/L; Na_2_HPO_4_ 1.63 g/L; NaCl 7.4 g/L), and centrifuged again under the same conditions twice. The precipitate was resuspended in 1 mL of sterile PBS, diluted at 1:100 in PBS, and then the quantity of yeast per inoculum was determined by counting in a Neubauer chamber before each experiment (Nikon Eclipse E2000 Microscope). The inoculum contained 1×10^4^ yeast/animal. Intratracheal injection (i.t.) of 1×10^4^ yeast/animal was performed with an inoculum of 30 μL/animal.

#### Anesthesia and analgesia

Prior to the surgical procedure, anesthesia and analgesia were performed by intraperitoneally (i.p.) administration of xylazine 0.2 mg/mL (10 mg/kg) and ketamine 20 mg/mL (100 mg/kg) in each animal.

#### Blood collection

Blood samples were collected from mice infected for 8, 10, 12, 14, and 16 days with R265. Mice were immobilized, and blood were collected by a small cut at the tail tip, allowing a drop to be collected for counting colony-forming units (CFU), before applying pressure with gauze to stop dripping and sterilizing with ethanol 70%. Undiluted blood, approximately 10 μL, were spread on petri dishes containing Sabouraud agar (20 g/L glucose (C_6_H_12_O_6_); 10 g/L peptone; 2% agar – Sigma, USA).

#### Survival, score analysis and animal weighing

A total of 10 animals per group were used for each experimental replicate (*n* = 10). After i.t injection of 1×10^4^ yeast/animal, mice were observed throughout the infection period, and deaths were recorded for survival analysis. Animal weighing was conducted before infection (day 0) and during the infection, twice a week. The percentage variation in weight relative to day 0 were represented in the results. The clinical features were assessed through a sum of scores for each animal. Evaluating the presence of ruffled fur, lethargy, hunched posture, tachypnea, and ataxia. Analysis was stopped when the first animal from each group died.

#### Organ fungal load

A total of 5 animals per group were used for each experimental replicate (*n* = 5). Lung, mediastinal lymph node, brain, and spleen were collected on days 8, 13, 16 and 18 post-infection (dpi) after intracardiac perfusion with approximately 20 mL of PBS in each animal. Each organ was weighed using an analytical balance. Subsequently, 100% of the mediastinal lymph node and 50% of the volume of each other organ was homogenized and diluted in 1 mL of sterile PBS in a Petri dish. The lung was diluted in 2 mL, and then 10 μL was spread onto a new Petri dish containing Sabouraud agar. For days 8 and 13, only the lung was diluted (1:1000), while for day 18 the lung was diluted (1:10000) and the dilution was (1:100) for the other organs. The plates were placed in an incubator at 30°C. Plate analysis was performed after 4 days by counting CFU. Lung photos were taken for representative image.

#### Airway responsiveness

A total of 5 animals per group were used for each experimental replicate (*n* = 5). Airway responsiveness was assessed as a change in airway function 24 h after the last challenge with aerosolized methacholine (Sigma-Aldrich) in a FinePoint R/C Buxcos Platform (Buxco Electronics, Sharon, CT, USA). Mice were anesthetized with ketamine (80 mg/kg). Neuromuscular activity was blocked with bromide pancuronium (1 mg/kg). Tracheostomized mice were mechanically ventilated, and lung function was assessed. The trachea was cannulated, and the cannula was connected to a pneumotachograph. Airflow and transpulmonary pressure were recorded using a Buxco Pulmonary Mechanics Processing System (Buxco Electronics, Wilmington, NC, USA). This instrument was used to calculate lung resistance (RL) (cm H_2_O/mL/s) in each breath cycle. Analogical signals from the computer were digitized using a Buxco Analog/Digital Converter (Buxco Electronics). Mice were allowed to stabilize for 5 min and increasing concentrations of methacholine (3, 9, 27 and 81 mg/mL) were aerosolized for 5 min each. Baseline pulmonary parameters were assessed with aerosolized phosphate-buffered saline (PBS). Expressed results comprised the mean absolute values of the responses of lung resistance collected during 5 min after the administration of methacholine aerosol.

#### Histopathology and cell size measurement

After euthanasia, lungs were collected from animals subjected to prior intracardiac perfusion with 20 mL of PBS. Halves of each lung was identified in cassettes and submerged in 10% formalin buffered with phosphate buffer (NaH_2_PO_4_ 0.538 g/L; Na_2_HPO_4_ 1.63 g/L) for 24 h, then in 70% ethanol for storage. Samples were subsequently collected for the dehydration process in successive baths of 90%, 100%, and 100% ethanol for 20 min each, followed by the clearing process in two baths with 100% xylene for 30 min each, and processed for embedding in paraffin. After embedding the sample in a paraffinized cassette using a microtome, the samples were cut into 5 μm thick sections and affixed to glass slides. Slides with histological sections undergone different staining techniques. Periodic Acid-Schiff (PAS) staining to visualize magenta-colored airway mucus, Masson’s Trichrome (MT) staining to visualize blue-colored collagen fibers, Picrosirius Red (PSR) staining to visualize carmine-colored collagen fibers,^83^ Harris hematoxylin and eosin Y (H&E) staining for the overall morphology of the organ, and modified alcian blue (MAB) at pH 2.5 for the identification of acidic polysaccharides present in the tissue, identifying the presence of fungal capsular polysaccharides.^84^ The slides with the sections were sealed with acrylic sealing using Entellan (Sigma-Aldrich). The analysis of the slides was performed using light microscopy on a scanning microscope (3DHISTECH Pannoramic MIDI II Digital Slide Scanner for H&E, PSR and MAB and Motic EasyScan Pro for H&E, MT and PAS). Image data analysis and cell size measurement were performed using the program (3DHISTECH Slide Viewer and ImageJ). Representative images were taken for each experimental group. For histopathological score analysis, the diffuse alveolar damage (DAD) score was evaluated based on the intensity and extent of alveolar collapse, alveolar inflammation, and alveolar edema, using a scale from 0 to 4, where 0 represents normal tissue and 4 indicates the worst prognosis, as assessed from H&E-stained slides. Peribronchial cell infiltration was quantified by counting the number of cell layers adjacent to the bronchoalveolar barrier, scored on a scale from 0 to 4, where 0 signifies no infiltration and 4 represents four or more layers. Collagen analysis on Masson's Trichrome (MT) slides was conducted using ImageJ software with the Color Deconvolution plugin. The blue channel was utilized to measure the percentage of blue-colored collagen fibers above a specified color threshold in the RGB image. Similarly, PAS slides were analyzed to assess magenta-colored mucus within bronchiolar airways, with the mean percentage of magenta expression calculated for each slide. For all analyses, 10 random areas per slide were examined to provide a comprehensive assessment for each animal at ×400 magnification. Representative images were taken and displayed at ×100 and ×630 magnification.

#### Cytokine assay

Cytokines from organ homogenates post-centrifugation at 1600 rpm/6 min were quantified using an enzyme-linked immunosorbent assay (ELISA), following the manufacturer’s protocols of (R&D Systems) for IL-1β ELISA kit, (PeproTech©) for IL-17A, IFN-γ, IL-13, IL-5, IL-4, IL-12, IL-22, TNF-α and IL-10 ELISA kits, and (ThermoFischer) for IL-33, IL-23 and TGF-β ELISA kits. The aliquoted homogenates were used for the quantification of the following cytokines. ELISA plates were pre-incubated separately the day before the analysis at 8°C with each respective capture antibodies. After blocking with bovine serum albumin (BSA) and washing five times with PBS-Tween 0.01%, aliquots of sample supernatants were incubated in the plates overnight. Subsequently, the plates were washed five times with PBS-Tween 0.01% and incubated with each respective biotinylated detection antibodies. After washing five times the plates, they were incubated with streptavidin-HRP, and washed five more times with PBS-Tween 0.01%. Finally, a reagent mixture of chromogen TMB with H_2_O_2_ was added, and the enzymatic reaction was stopped with phosphoric acid until a bluish color appeared. The resultant yellowish color was measured at 450 nm in an ELISA microplate reader SpectraMaxE5. Cytokine concentrations were determined using a standard curve of recombinant cytokines.

#### Flow cytometry

Homogenates from lungs were centrifuged to collect supernatant for ELISA assays. The lung precipitate was resuspended in collagenase type VIII (Sigma Aldrich) at 5 mg/mL in PBS at 37°C under agitation for 60 min to dissociate the tissue matrix and obtain the cell suspension. To stop the reaction, 10% fetal bovine serum (FBS) were added to the solution. Homogenate samples were washed with PBS, centrifuged, and placed in RPMI culture medium (5 mM Glucose) supplemented with 10% FBS. Samples were then incubated in the presence of activators PMA (5 ng/mL) and ionomycin (1 μM) for 6 h. To inhibit cytokine secretion, monensin (2 μM) (GolgiStop) was also added for 6 h. After incubation, lung samples were used in one cytometry panel to evaluate the ratio of lung cell populations. It used the following fluorochrome-conjugated antibodies: Live/Dead (BV510), anti-CD45 (BV421), anti-CD3 (APC-Cy7), anti-F4/80 (APC), anti-CD11b (PE-Cy7), anti-Ly6C (FITC), anti-Ly6G (BV650) and anti-SiglecF (PE). This panel consists of the identification of leukocytes (Live/Dead^−^CD45^+^), T lymphocytes (Live/Dead^−^CD45^+^CD3^+^), infiltrating macrophages (IM) (Live/Dead-CD45^+^F4/80^+^CD11b^+^SiglecF^−^), neutrophils (Live/Dead^−^CD45^+^F4/80^−^CD11b^+^Ly6G^+/−^Ly6C^−^), and eosinophils (Live/Dead^−^CD45^+^F4/80^−^CD11b^+^Ly6G^−^SiglecF^+^). Differently, another panel was developed for identifying IL-22 cytokine producing cells, as well other cell activation cocktail with Brefeldin A (BioLegend©). Therefore, the following fluorochrome-conjugated antibodies were used: anti-CD45 (BV786), anti-CD19 (BV711), anti-TCRγδ (AF488), anti-TCRβ (PE-Cy7), anti-NK1.1 (APC-Cy7), anti RORγt (AF647) and anti-IL-22 (PE). CD45^−^IL-22^+^ (IL-22^+^ non-leucocytes), CD45^+^IL-22^+^ (IL-22^+^ leukocytes), CD45^+^CD19^+^IL-22^+^ (IL-22 B cells), CD45^+^CD19^−^TCRβ^−^TCRγδ^+^IL-22^+^ (IL-22^+^ γδT cells), CD45^+^CD19^−^TCRβ^+^TCRγδ^−^IL-22^+^ (IL-22^+^ αβT cells), CD45^+^CD19^−^TCRβ^−^TCRγδ^−^NK1.1^+^IL-22^+^ (IL-22^+^ NK cells), CD45^+^CD19^−^TCRβ^−^TCRγδ^+^NK1.1^−^RORγ^+^IL-22^+^ (IL-22^+^ ILC3s), CD45^+^CD19^−^TCRβ^−^TCRγδ^+^NK1.1^−^RORγ^−^IL-22^+^ (IL-22^+^ non-lymphoid leukocytes). The following cell populations were evaluated: Samples were first incubated with FcBlock (CD16/CD32) (1:200) in 10% FBS and 5% murine serum in PBS for 20 min. They were then incubated with the mixture of antibodies for 20 min, except for the unmarked control of the lung cell suspension. Antibodies were diluted (1:200). After washing with FACS buffer (PBS +1% FBS +0.002% azide), centrifuged samples were fixed with fixation solution (Fix/Perm) (BD) for 15 min, washed, and centrifuged with permeabilization buffer (Perm/Wash) (BD). They were then incubated again with Perm/Wash for 20 min. After washing and centrifuging again with Perm/Wash, samples were stored in FACS buffer protected from light at 8°C for up to 1 week. Compensation controls for the antibodies were prepared with single-stained samples using the same process described above. Sample reading was done by acquiring 100,000 events per sample tube on a flow cytometer (BD LSRFortessa X-20 Cell Analyzer), and data analysis were performed using the program (FlowJo VX).

### Quantification and statistical analysis

Statistical analyses were performed using GraphPad Prism 9.5.0. The t-Student test or Mann–Whitney U test was employed for comparisons between two groups, and the Log rank test (Mantel-Cox test) were used for survival curve comparisons between two groups. For comparisons involving multiple groups of animals, an analysis of variance (ANOVA) or Kruskal-Wallis test was conducted, followed by post-tests such as Holm-Sidak or Dunn, respectively. Significance was indicated by *p*-values <0.05, with specific symbols denoting different levels of statistical significance: ∗∗∗∗ (*p* < 0.0001), ∗∗∗ (*p* < 0.001), ∗∗ (*p* < 0.01), ∗ (*p* < 0.05), or ^####^ (*p* < 0.0001), ^###^ (*p* < 0.001), ^##^ (*p* < 0.01), ^#^ (*p* < 0.05).
