# Supplementary material for: IL-22 and IL-23 regulate the anticryptococcal response during Cryptococcus deuterogattii infection
Source: iScience. 2024 Sep 27;27(10):111054. doi: 10.1016/j.isci.2024.111054 (PMC11615251; doi:10.1016/j.isci.2024.111054)
Supplement: Document S1. Figures S1–S7 [file mmc1.pdf]

iScience, Volume *27*

Supplemental information

IL-22 and IL-23 regulate

the anticryptococcal response

during *Cryptococcus deuterogattii* infection

Israel Diniz-Lima, Ariel Gomes, Mayck Medeiros, Joyce Cristina Guimarães-de-Oliveira, Idália Maria Ferreira-dos-Santos, Elias Barbosa da Silva-Junior, Alexandre Morrot, Danielle Oliveira Nascimento, Leonardo Freire-de-Lima, Lycia de Brito-Gitirana, Fernanda Ferreira Cruz, Debora Decote-Ricardo, Herbert Leonel de Matos Guedes, and Celio Geraldo Freire-de-Lima

Supplemental information

Supplemental Figures S1-S7

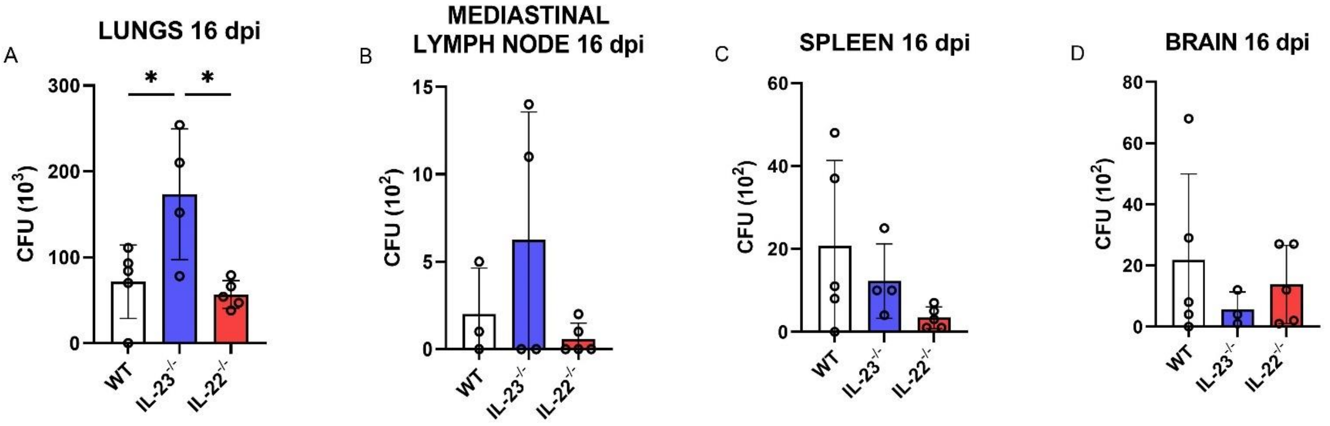

Figure S1. Colony-forming unit analysis from organs 16 days post-infection, Related to Figure 1. Wild-type (WT) (n=5), IL-23$^{-/-}$ (n=5) and IL-22$^{-/-}$ (n=5) C57BL/6 mice were intratracheally (i.t.) infected with 10^4 R265 yeasts. (A-D) Animals had their organs surgically collected after euthanasia at 16 dpi and were macerated at different dilutions. Lung samples were diluted at 1:1000, while other organs were diluted at 1:100. Diluted samples were plated onto Sabouraud-agar culture plates for colony-forming units (CFU) to be counted 4 days later. Fungal burden from (A) Lung, (B) Mediastinal lymph node, (C) Spleen and (D) Brain. * $p < 0.05$ for comparison between groups. Data are represented as mean +/- SD.

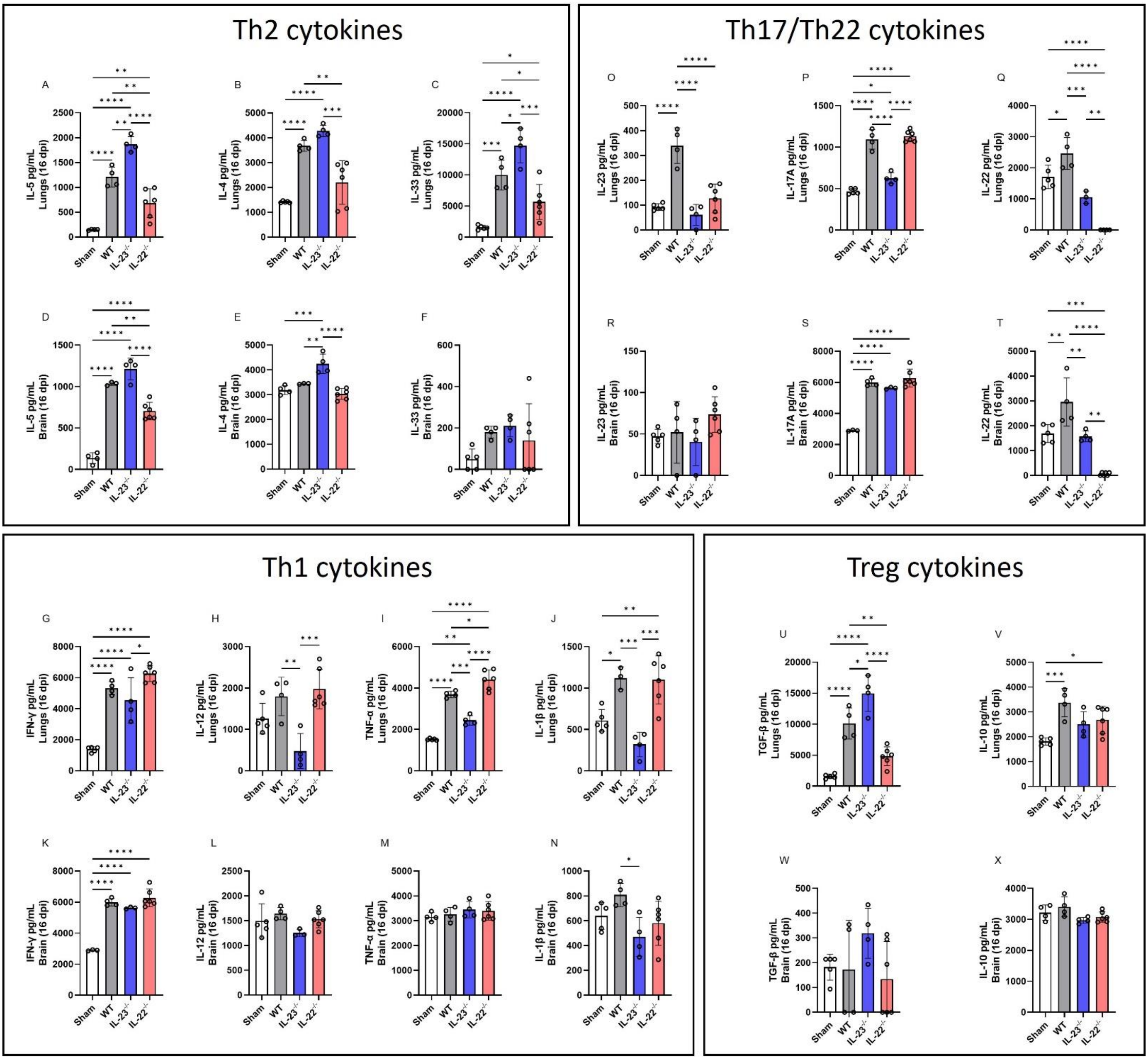

Figure 2. Cytokine analysis of lung and brain tissues in mice 16 days post-infection, Related to Figure 2. Wild-type (WT) (n=5), IL-23$^{-/-}$ (n=5) and IL-22$^{-/-}$ (n=5) C57BL/6 mice were intratracheally (i.t.) infected with 10^4 R265 yeast and observed over time. A WT sham group (Sham) (n=5) inoculated i.t. with PBS vehicle was used. Sham animals were euthanized after the assay. Animals had their lungs and brain surgically collected after euthanasia at 16 dpi. Cytokine analysis from (A-C, G-J, O-Q, U and V) lungs and (D-F, K-N, R-T, W and X) brain 16 dpi, (A and D) IL-5, (B and E) IL-4, (C and F) IL-33, (G and K) IFN-γ, (H and L) IL-12, (I and M) TNF-α, (J and N) IL-1β, (O and R) IL-23, (P and S) IL-17A, (Q and T) IL-22, (U and W) TGF-β and (V and X) IL-10 were measured by ELISA. **** $p < 0.0001$, *** $p < 0.001$, ** $p < 0.01$, * $p < 0.05$ for comparisons between groups. Data are represented as mean +/- SD.

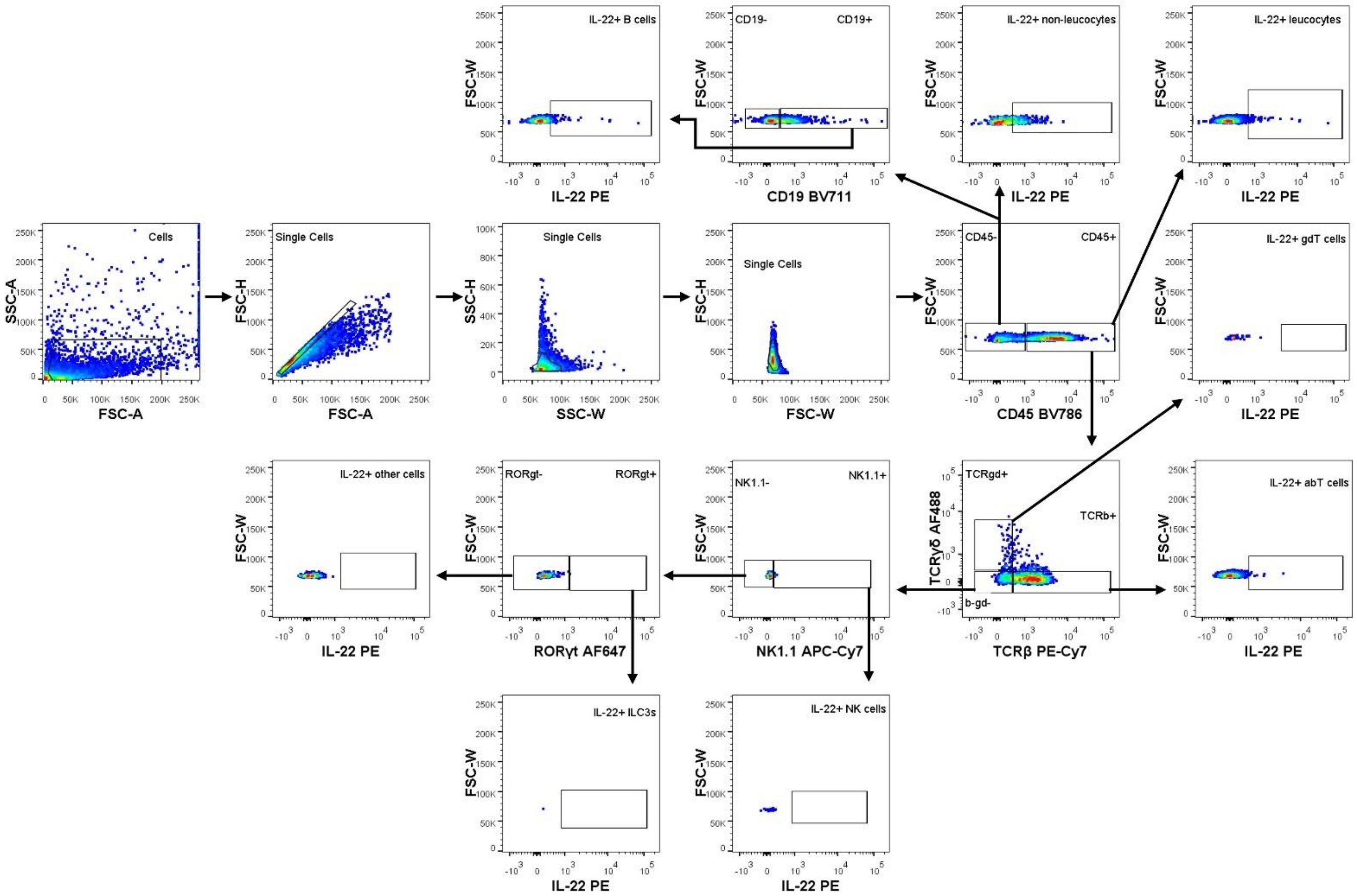

Figure S3. Flow cytometry gating strategy employed to analyze IL-22 cell contributions in the lungs, Related to Figure 3. The flow cytometry gating strategy consisted initially in a sequence of single cells restriction gates from the SSC-A×FSC-A gate as follow: single cell 1 (FSC-H×FSC-A), single cell 2 (SSC-H×SSC-W), single cell 3 (FSC-H×FSC-W). After the single cell analysis several IL-22$^+$ cell population were analyzed: CD45$^-$IL-22$^+$ (IL-22$^+$ non-leucocytes), CD45$^+$IL-22$^+$ (IL-22$^+$ leucocytes), CD45$^+$CD19$^+$IL-22$^+$ (IL-22 B cells), CD45$^+$CD19$^-$TCRβ$^-$TCRγδ$^+$IL-22$^+$ (IL-22$^+$ γδT cells), CD45$^+$CD19$^-$TCRβ$^+$TCRγδ$^-$IL-22$^+$ (IL-22$^+$ αβT cells), CD45$^+$CD19$^-$TCRβ$^-$TCRγδ$^-$NK1.1$^+$IL-22$^+$ (IL-22$^+$ NK cells), CD45$^+$CD19$^-$TCRβ$^-$TCRγδ$^+$NK1.1$^-$RORγ$^+$IL-22$^+$ (IL-22$^+$ ILC3s), CD45$^+$CD19$^-$TCRβ$^-$TCRγδ$^+$NK1.1$^-$RORγ$^-$IL-22$^+$ (IL-22$^+$ non-lymphoid leucocytes). Arrows indicate the sequence of inclusion gates.

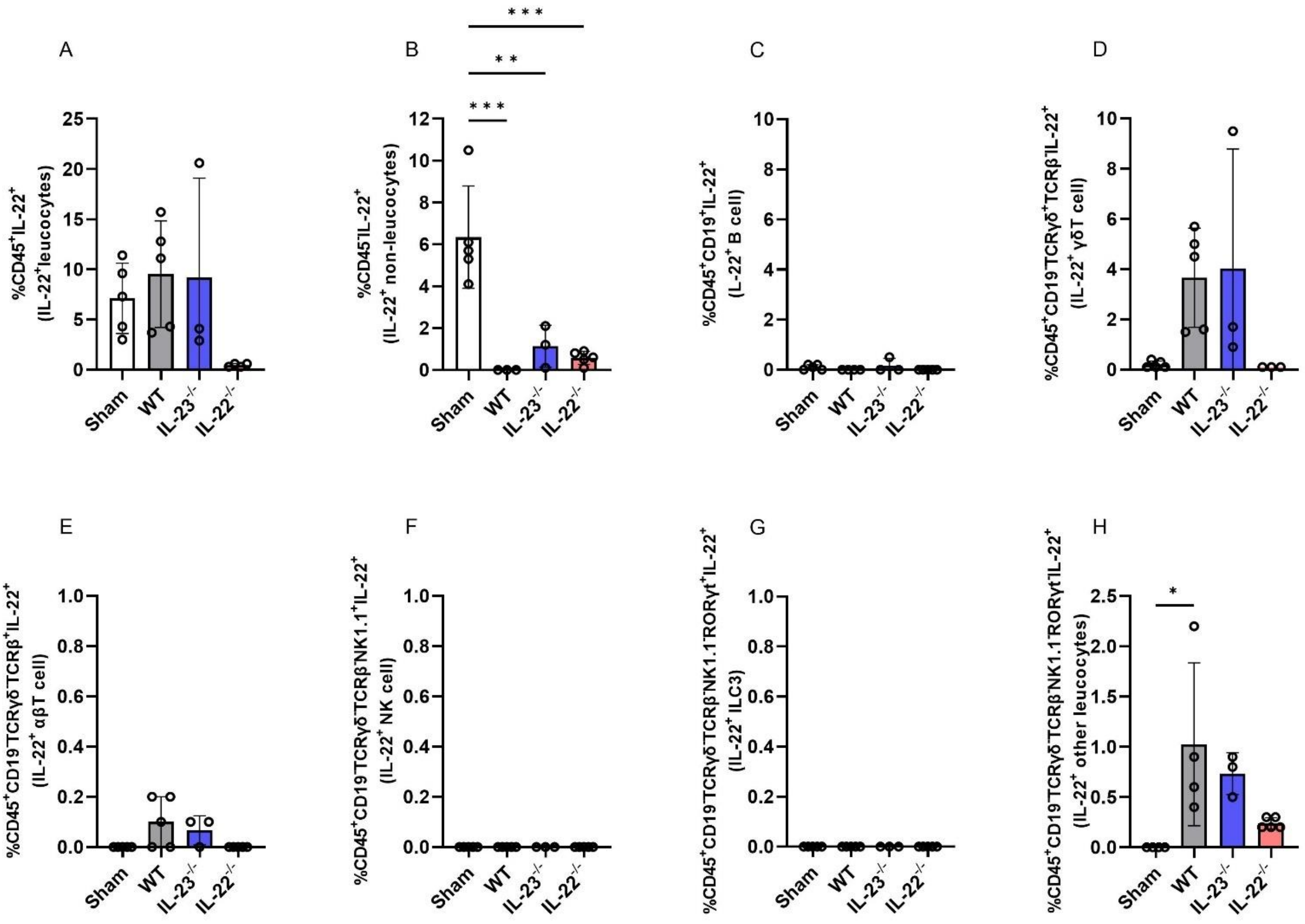

Figure S4. Analysis of lung IL-22 cell contributions in mice 16 days post-infection, Related to Figure 3. Wild-type (WT) (n=5), IL-23$^{-/-}$ (n=5) and IL-22$^{-/-}$ (n=5) C57BL/6 mice were intratracheally (i.t.) infected with 10^4 R265 yeast. A WT sham group (Sham) (n=5) inoculated i.t. with PBS vehicle was used. Sham animals were euthanized after the assay. Animals had their lungs surgically collected after euthanasia at 16 dpi, macerated and cell suspension acquired. Single cell suspensions were stained for cytometry analysis. (A) CD45$^+$IL-22$^+$ (IL-22$^+$ leucocytes), (B) CD45$^-$IL-22$^+$ (IL-22$^+$ non-leucocytes), (C) CD45$^+$CD19$^+$IL-22$^+$ (IL-22$^+$ B cells), (D) CD45$^+$CD19$^-$TCRβ$^-$TCRγδ$^+$IL-22$^+$ (IL-22$^+$ γδT cells), (E) CD45$^+$CD19$^-$TCRβ$^+$TCRγδ$^-$IL-22$^+$ (IL-22$^+$ αβT cells), (F) CD45$^+$CD19$^-$TCRβ$^-$TCRγδ$^-$NK1.1$^+$IL-22$^+$ (IL-22$^+$ NK cells), (G) CD45$^+$CD19$^-$TCRβ$^-$TCRγδ$^+$NK1.1$^-$RORγ$^+$IL-22$^+$ (IL-22$^+$ ILC3s), (H) CD45$^+$CD19$^-$TCRβ$^-$TCRγδ$^+$NK1.1$^-$RORγ$^-$IL-22$^+$ (IL-22$^+$ non-lymphoid-leucocytes). *** $p < 0.001$, ** $p < 0.01$ for comparisons between groups. Data are represented as mean +/- SD.

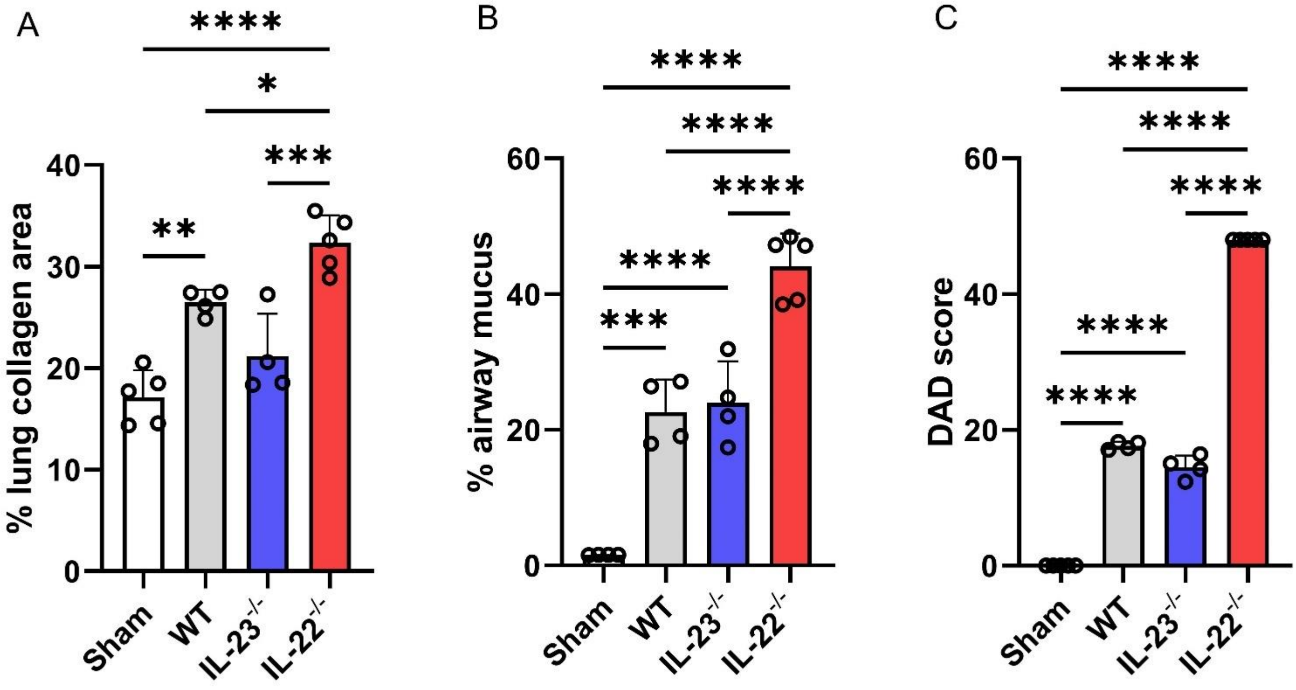

Figure S5. Histopathological scoring analysis of lung parenchyma in mice 16 days post-infection, Related to Figure 5. Wild-type (WT) (n=5), IL-23$^{-/-}$ (n=5) and IL-22$^{-/-}$ (n=5) C57BL/6 mice were intratracheally (i.t.) infected with 10^4 R265 yeasts. A WT sham group (Sham) (n=5) inoculated i.t. with PBS vehicle was used. Sham animals were euthanized after the assay. Animals had their lungs surgically collected after euthanasia at 16 dpi and taken for hematoxylin and eosin (H&E) staining, Masson's Trichrome staining and Periodic Acid–Schiff (PAS) staining. (A) percentage of lung parenchyma collagen area after color deconvolution analysis in ImageJ software, (B) percentage of airway mucus area after color deconvolution analysis in ImageJ software, (C) Diffuse Alveolar Damage (DAD) score on H&E histopathological samples. **** $p < 0.0001$, *** $p < 0.001$, ** $p < 0.01$, * $p < 0.05$ for comparisons between groups. Data are represented as mean +/- SD.

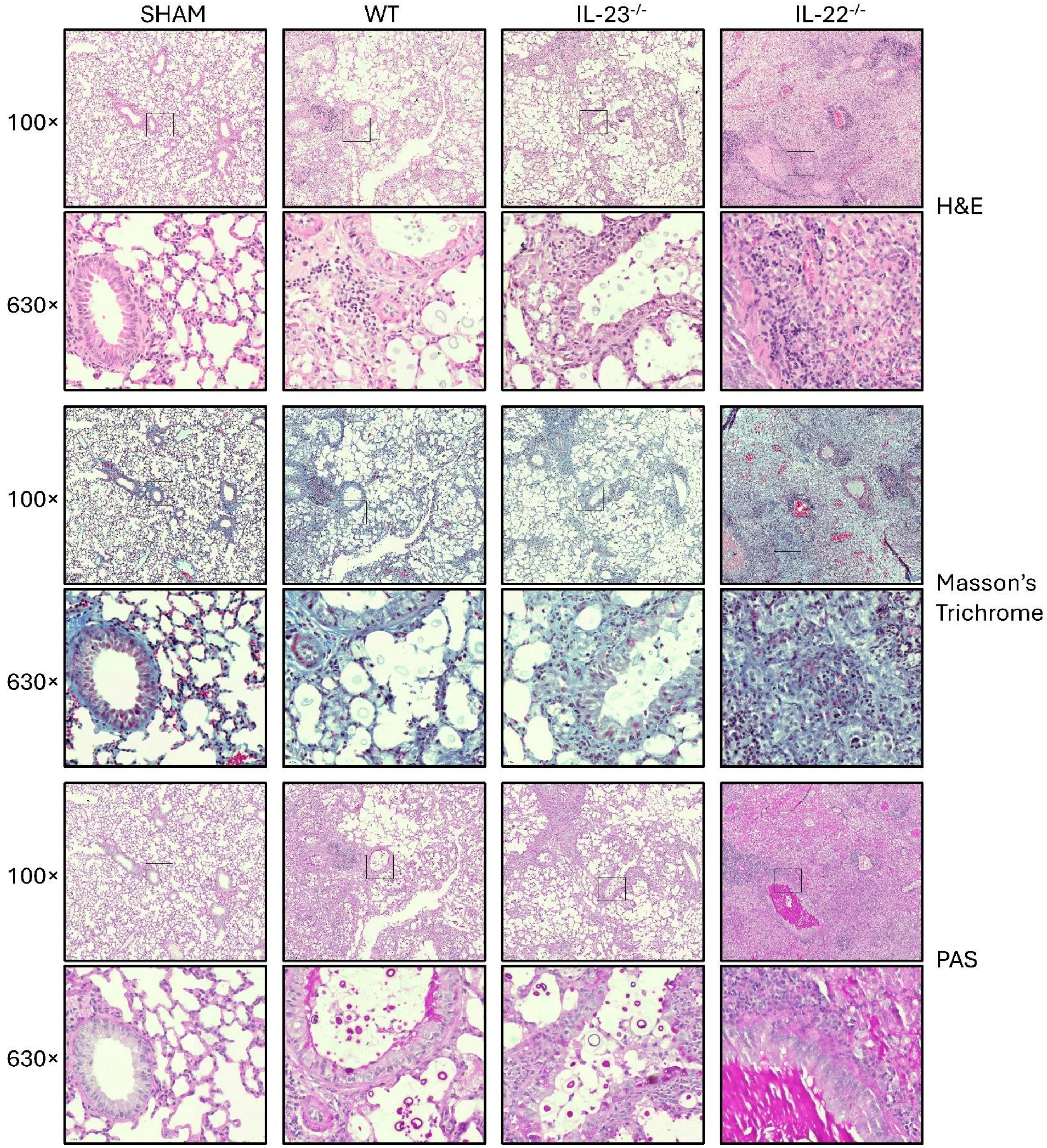

Figure S6. Representative images showcasing the histopathological analysis of lung parenchyma in mice 16 days post-infection, Related to Figure 5. Wild-type (WT) (n=5), IL-23$^{-/-}$ (n=5) and IL-22$^{-/-}$ (n=5) C57BL/6 mice were intratracheally (i.t.) infected with 10^4 R265 yeasts. A WT sham group (Sham) (n=5) inoculated i.t. with PBS vehicle was used. Sham animals were euthanized after the assay. Lung tissues were surgically collected post-euthanasia at 16 days post-infection (dpi), followed by histological processing for Hematoxylin and Eosin (H&E) staining, Masson's Trichrome (MT) staining, and Periodic Acid-Schiff (PAS) staining. The images are presented in pairs, with ×100 magnification views paired with corresponding ×630 magnification images. The ×630 magnified images are derived from the regions indicated by small squares within the ×100 magnified fields. Each set of images represents the same field for each staining method across all experimental groups. H&E staining highlights cellular structures, Masson's Trichrome staining identifies collagen fibers in blue, and PAS staining reveals mucus in magenta within the airways.

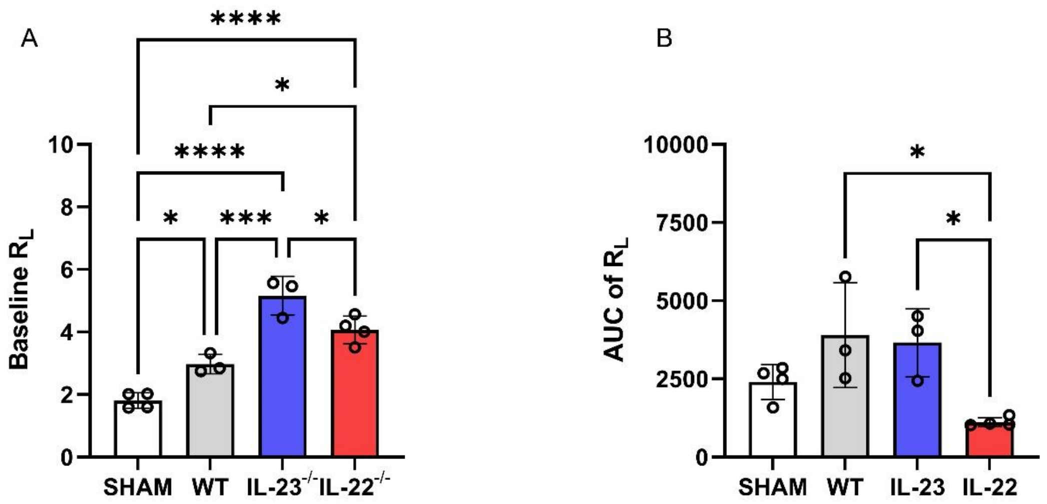

Figure S7. Lung resistance of mice 16 days post-infection, Related to Figure 6. Wild-type (WT) (n=5), IL-23$^{-/-}$ (n=5) and IL-22$^{-/-}$ (n=5) C57BL/6 mice were intratracheally (i.t.) infected with 10^4 R265 yeasts. A WT sham group (Sham) (n=5) inoculated i.t. with PBS vehicle was used. Sham animals were euthanized after the assay. Animals were anesthetized and placed in a ventilation chamber. Methacholine (Sigma-Aldrich) is nebulized in the chamber at concentrations of 3, 9, 27, and 81 µg/mL to induce airway constriction. Airway resistance is measured using a plethysmograph. (A) the analysis includes measurement of baseline resistance without methacholine and (B) the area under the curve (AUC) of the responses to methacholine treatment to assess airway reactivity. **** $p < 0.0001$, *** $p < 0.001$, ** $p < 0.01$, * $p < 0.05$ for comparisons between groups. Data are represented as mean +/- SD.
